# Ataxia and cerebellar hypoexcitability in a mouse model of *SCN1B*-linked Dravet syndrome

**DOI:** 10.1172/jci.insight.187606

**Published:** 2025-09-09

**Authors:** Yukun Yuan, Heather A. O’Malley, Jesse J. Winters, Alfonso Lavado, Nicholas S. Denomme, Shreeya Bakshi, Samantha L. Hodges, Luis Lopez-Santiago, Chunling Chen, Lori L. Isom

**Affiliations:** 1 Department of Pharmacology, University of Michigan Medical School, Ann Arbor, Michigan, USA.; 2 Center for Pediatric Neurological Disease, Pediatric Translational Neuroscience Initiative, Department of Cellular and Molecular Biology, St. Jude Children’s Research Hospital, Memphis, Tennessee, USA.

**Keywords:** Genetics, Neuroscience, Epilepsy, Mouse models, Sodium channels

## Abstract

Patients with Dravet syndrome (DS) present with severe, spontaneous seizures and ataxia. While most patients with DS have variants in the sodium channel Nav1.1 α subunit gene, *SCN1A*, variants in the sodium channel β1 subunit gene, *SCN1B*, are also linked to DS. *Scn1b* null mice model DS, with spontaneous generalized seizures that start in the second week of life. In *Scn1b* null cerebellum, neuronal pathfinding is severely altered, and Purkinje cells (PCs) and granule neurons have altered excitability. Here, we show that *Scn1b* null mice are ataxic. Expression of β1 protein in WT cerebellum, assessed using a CRISPR transgenic mouse model containing an in-frame V5 epitope tag at the β1 C-terminus, is widespread. *Scn1b* null PCs and interneurons in cerebellar slices have increased thresholds for action potential initiation and decreased repetitive firing frequency compared with WT. *Scn1b* null PCs have reduced transient and resurgent sodium current densities. We propose that reduced PC excitability underlies the ataxic phenotype of *Scn1b* mice. In addition, because cerebellar output to other areas of the brain can result in termination of seizures, we propose that PC hypoexcitability exacerbates the severe phenotype of this mouse model.

## Abstract 2

Yuan and colleagues use a transgenic mouse model of Dravet syndrome to study the contributions of altered neuronal excitability in the cerebellum to ataxia.

## Graphical abstract

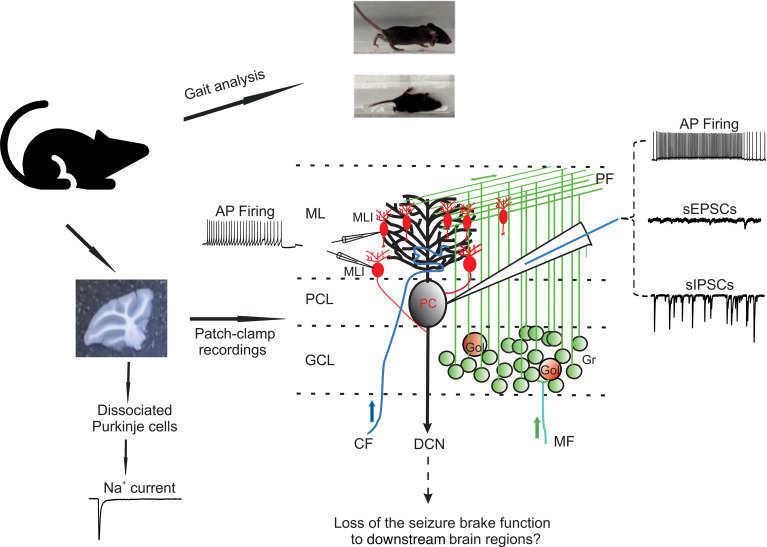

## Introduction

Dravet syndrome (DS) is a devastating developmental and epileptic encephalopathy (DEE) that presents in childhood (1). Patients with DS have severe, often intractable, seizures; behavioral and developmental delays; profound intellectual disability; and ataxia. The majority of patients with DS have variants in the gene *SCN1A* that result in haploinsufficiency of the voltage-gated sodium channel (VGSC) Nav1.1 α subunit (2, 3). A smaller cohort of patients with DS have biallelic variants in the VGSC β1 subunit gene, *SCN1B*. *SCN1B* variants are also linked to the more severe early infantile DEE (OMIM term DEE52) (4–9). Non-pore-forming β1 subunits function as channel modulators and chaperones to the plasma membrane as well as immunoglobulin superfamily cell adhesion molecules (CAMs) that interact with other β1 subunits on adjacent cells, with other CAMs, or with extracellular matrix molecules (6–9). *Scn1b* null mice model DEE52, with generalized seizures beginning in the second week of life and sudden unexpected death in epilepsy (SUDEP) in 100% of mice prior to weaning (10, 11).

The cerebellum is essential for proper motor control in mammals (12). The cerebellum also plays critical roles in executive function and in seizure networks and thus may offer therapeutic targets for intellectual disability and seizure control (13–18). Our previous body of work demonstrated that *Scn1b* plays a key role in cerebellar development in mice (19–22). However, the role of *Scn1b* in cerebellar excitability and network communication in vivo has not been described to our knowledge. Here, we studied the expression of VGSC β1 subunits in mouse cerebellum as well as the outcome of *Scn1b* deletion on cerebellar excitability in neonatal mice. Our results show that *Scn1b* null mice are ataxic. GABAergic neurons in the *Scn1b* null cerebellar cortex are overall hypoexcitable, and *Scn1b* null Purkinje cells (PCs) have significantly reduced transient and resurgent sodium current densities. We propose that proper *Scn1b* expression in the cerebellum is essential for motor control and that *Scn1b* deletion contributes to cerebellar ataxia and seizure severity.

## Results

### Scn1b null pups are ataxic.

**Figure 1:**
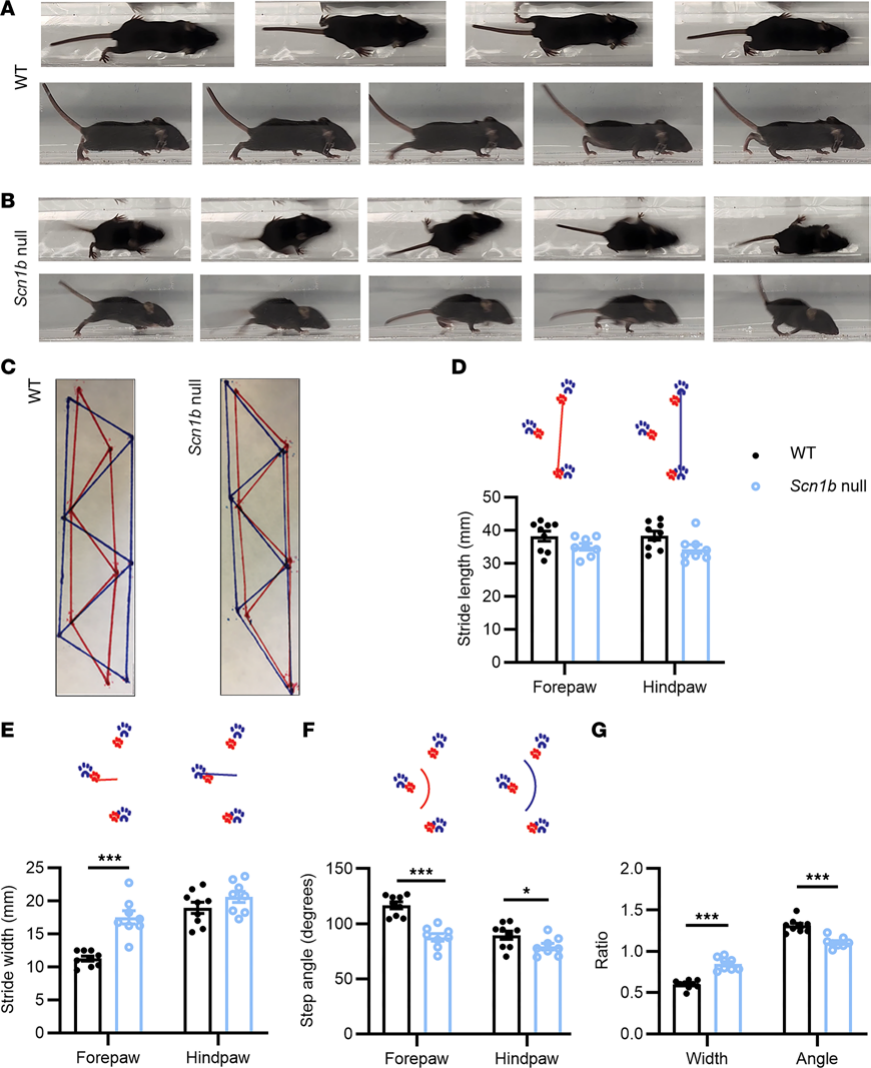
*Scn1b* null mice are ataxic. *Scn1b* null mice display multiple stride differences compared with WT. (**A** and **B**) Images of a single stride captured from video recordings of 1 WT (**A**) and 1 *Scn1b* null (**B**) mouse. Top: Top-down view; bottom: side view. (**C**) Annotated footprints from 3 consecutive strides of 1 WT (left) and 1 *Scn1b* null (right) mouse. Insets in **D**–**F** display the measurement locations for each analysis type. (**D**) Stride length for forepaw and hind paw steps. (**E**) Stride width for forepaw and hind paw steps. (**F**) Step angle for forepaw and hind paw steps. (**G**) Ratio of step width and step angle. WT: filled circles; *Scn1b* null: unfilled circles. *P < 0.05; ***P < 0.001 (unpaired *t* test, 2-tailed).

We performed gait analysis on *Scn1b* null and wild-type (WT) littermate pups. We performed these tests at P16 to avoid SUDEP, which begins around P18 in this model (10). The gaits of 3 null and 3 WT pups were recorded using top-down and side view videos (Figure 1, A and B, and Supplemental Videos 1 and 2; supplemental material available online with this article; https://doi.org/10.1172/jci.insight.187606DS1). We also performed footprint analysis to quantify differences between genotypes (23) (Figure 1C). Gait analysis parameters are summarized in Supplemental Table 1. Pups were allowed to walk freely along a narrow acrylic path to imprint painted red forepaw prints and blue hind paw prints on filter paper. Stride length, stride width, and step angle were measured from 8 *Scn1b* null and 9 WT pups. Stride lengths, measured from either hind limb prints (*Scn1b* null, 34.3 ± 1.32 mm vs. WT, 38.4 ± 1.36 mm; *P* = 0.577) or forelimb prints (*Scn1b* null, 35.0 ± 1.00 mm vs. WT, 38.3 ± 1.51 mm; *P* = 0.451), were not different between genotypes (Figure 1D). In contrast, *Scn1b* null pups had greater forepaw, but not hind paw, stride widths compared with WT (*Scn1b* null forepaw, 17.5 ± 1.00 mm vs. WT forepaw, 11.3 ± 0.38 mm; *P* < 0.001; *Scn1b* null hind paw, 20.6 ± 0.85 mm vs. WT hind paw, 18.9 ± 0.86 mm; *P* = 0.19). *Scn1b* null pups walked with their forepaws nearly at the same width as their hind paws. In contrast, WT pups walked with a wider hind paw than forepaw stance (Figure 1E). The ratio between forepaw and hind paw widths was larger for *Scn1b* null pups compared with WT (*Scn1b* null ratio, 0.85 ± 0.03; WT ratio, 0.60 ± 0.02; *P* < 0.001). Data are shown as mean ± SEM.

Because the growth of *Scn1b* null pups plateaus after approximately P10 (10, 24), we dissected and measured the lengths of the humerus and femur bones from 6 pups of each genotype and incorporated these differences into the analyses of forelimb and hind limb gait, respectively. Bone lengths were shorter in *Scn1b* null pups compared with WT. *Scn1b* null femurs were 8% shorter (*Scn1b* null, 9.04 ± 0.23 mm vs. WT, 9.79 ± 0.14 mm; *P* = 0.019). *Scn1b* null humeri were 5% shorter than WT (*Scn1b* null, 8.20 ± 0.14 mm vs. WT, 8.67 ± 0.06 mm; *P* = 0.011). We normalized the data for stride widths to the relevant limb bone to obtain a more accurate measure of the positioning of the limbs during walking. *Scn1b* null pups had a significantly wider forepaw stance, adjusted for humerus length (*Scn1b* null, 1.64 ± 0.09, *n* = 8 vs. WT, 1.00 ± 0.03, *n* = 9; *P* < 0.001), and hind paw stance, adjusted for femur length (*Scn1b* null, 1.18 ± 0.05, *n* = 8 vs. WT, 1.00 ± 0.05, *n* = 9; *P* = 0.016).

We also measured the angle formed by 3 consecutive steps made by each mouse (Figure 1F). In agreement with the observed difference in width of forepaw and hind paw stances between genotypes, we found significantly different angles of forepaw steps (*Scn1b* null, 87.75° ± 3.39°, *n* = 8 null vs. WT, 116.75° ± 3.08°, *n* = 9; *P* < 0.001) and hind paw steps (*Scn1b* null, 79.25° ± 2.93°, *n* = 8 vs. WT, 89.61° + 3.72°, *n* = 9; *P* = 0.048). *Scn1b* null forepaw and hind paw step angles were more similar to one another compared with WT step angles. Consequently, the ratio between the forepaw angle and the hind paw angle was higher for WT than for null pups (*Scn1b* null, 1.11 ± 0.23, *n* = 8 vs. WT, 1.31 ± 0.028, *n* = 9; *P* < 0.001) (Figure 1G). Taken together, footprint analyses showed a significant difference in gait parameters between genotypes at P16, suggesting that *Scn1b* deletion impacts cerebellar motor function in neonatal animals.

### Sodium channel β1 subunits are widely expressed in mouse cerebellum.

To facilitate the localization of VGSC β1 subunits in WT mouse cerebellum using high-affinity, commercially available antibodies, we generated a CRISPR transgenic knockin mouse line expressing an in-frame V5 epitope tag at the β1 C-terminus (Supplemental Figure 1, A and B). We observed that β1-V5 mice live normal life spans, breed normally, and have no overt phenotype, suggesting that the V5 epitope tag does not impact β1 protein function. We stained cerebellar sections of homozygous P19 β1-V5 mice with anti-V5 antibody to label β1 subunits, anti-NeuN to label neuronal cell bodies, and anti-AnkG antibody to label axon initial segments (AISs) (Supplemental Figure 1C). We observed dense anti-V5 staining in the cerebellar molecular layer (ML, shown in cyan), suggesting wide expression of β1 subunits in PC dendrites, basket cells, stellate cells, and parallel fibers (PFs). Less dense anti-V5 staining was observed in the granule cell layer, consistent with our previous work showing anti-β1 antibody staining in cultured cerebellar granule neuron (CGN) soma and axons as well as in PC soma, ML, CGNs, and Bergmann glia in cerebellar slices (19, 20) (Supplemental Figure 1C, panel i, cyan). We also observed colocalization of anti-V5 and anti-AnkG staining at PC AISs, consistent with our previous work (20) (Supplemental Figure 1C, panel ii, cyan).

### Scn1b null PCs are hypoexcitable with reduced transient and resurgent sodium current (I_Na_) density.

**Figure 2:**
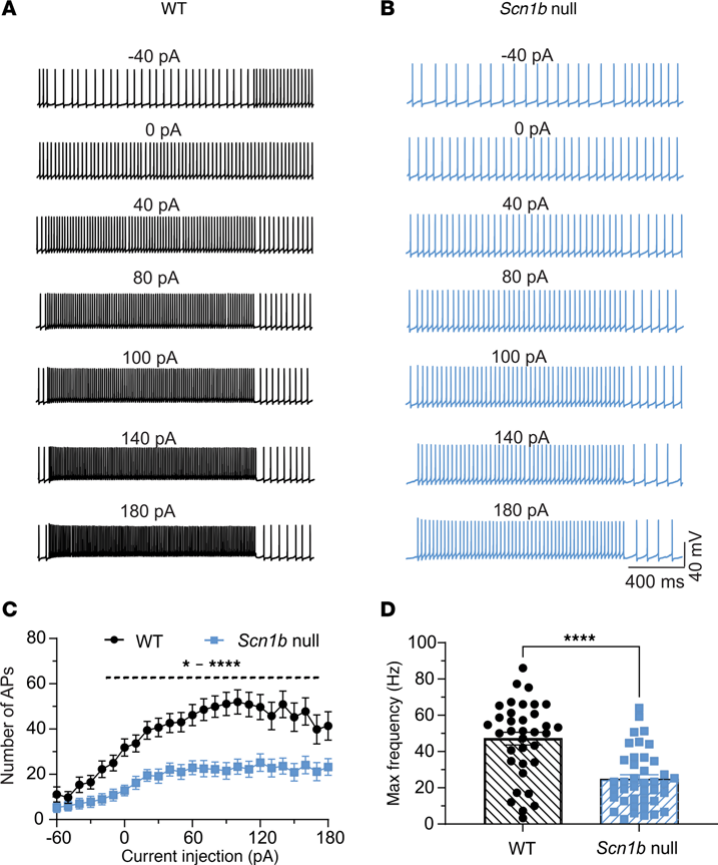
*Scn1b* null PCs are hypoexcitable. (**A** and **B**) Representative traces showing evoked repetitive firing of PCs in cerebellar slices from WT (**A**) or null (**B**) mice. Repetitive AP firing was evoked by injections of 1,500 ms pulse currents of –60 pA to +180 pA (only selected –40 pA– to 180 pA–evoked responses are shown). (**C**) Input-output curves of AP firing for WT (black) and null (blue) PCs in response to current injections from –60 pA to 180 pA. Null PCs show reduced AP firing frequencies at all stimulation intensities. Note, cells firing 0 APs or 3 or fewer APs are not included in input-output analyses. The dotted line indicates the range of -20 pA to 180 pA current points. (**D**) Null PCs show decreased maximal firing frequencies. Values are mean ± SEM of 36 cells from 23 WT mice or 41 cells from 29 null mice, respectively. *P < 0.05, ****P < 0.0001, * ^–^ **** represent P values from <0.05 to <0.0001 (2-way ANOVA for **C**, unpaired *t* test for **D**, 2-tailed *P* value).

The ataxic phenotype of *Scn1b* null mice suggested altered cerebellar neuron excitability. To test this hypothesis, we first measured action potential (AP) firing rates in PCs using whole-cell current clamp recording in response to injected currents ranging from –60 pA to 180 pA. PC axons are the sole output of the cerebellar cortex, and thus any changes in the function of upstream neurons can be detected as changes in PC excitability. Representative traces are shown in Figure 2, A and B. WT PCs showed sustained tonic firing of single APs that increased in frequency with increased injection of current (Figure 2A, black). In contrast, *Scn1b* null PCs had a marked reduction in firing frequency across the range of depolarizing currents injected (Figure 2B, blue). Input-output curves for both genotypes are quantified in Figure 2C, showing reduced AP firing frequencies for null PCs at all stimulation intensities. In addition, the maximal firing frequency for null PCs was significantly reduced compared with WT (Figure 2D).

**Figure 3:**
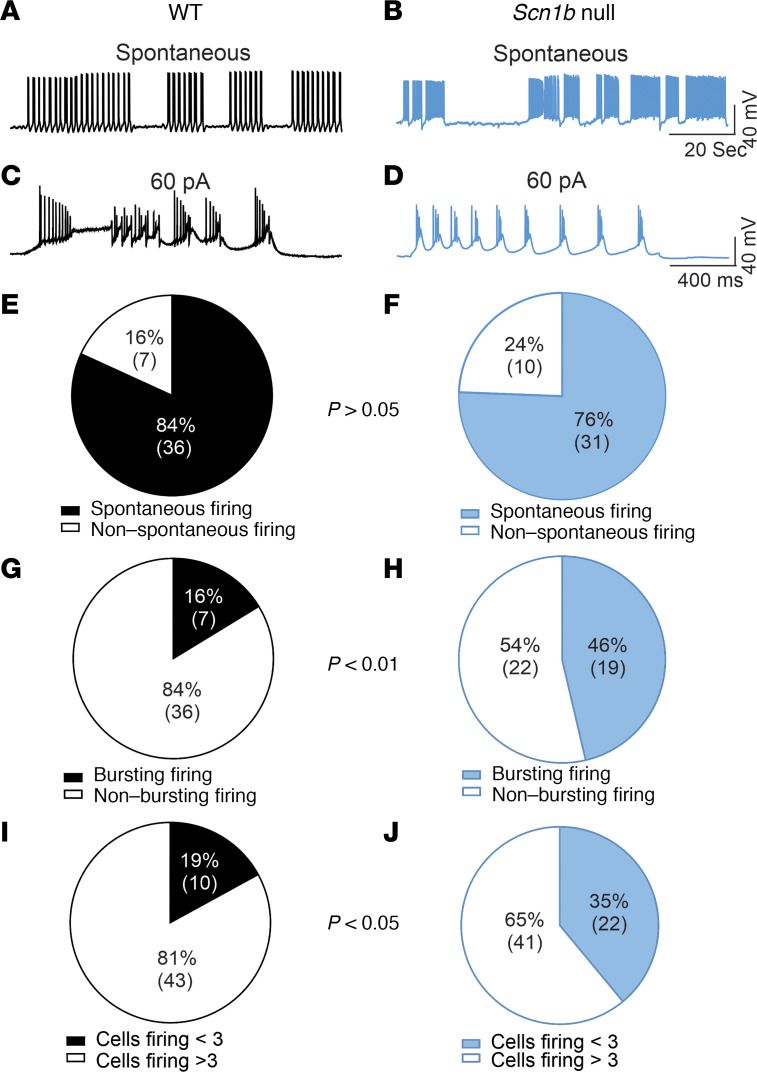
*Scn1b* null PCs show aberrant bursting activity. (**A**–**D**) Representative examples of spontaneous (**A** and **B**) or evoked (**C** and **D**) burst firing from WT (**A** and **C**) or null (**B** and **D**) PCs under current-clamp. Traces are representative of 7 cells of 43 WT PCs (23 mice) or 19 cells of 41 *Scn1b* null PCs (29 mice). (**E** and **F**) Rates of spontaneous firing for WT (black) or null (blue) PCs. Values in parentheses represent numbers of cells that did or did not fire spontaneously, as indicated. Differences between genotypes are not significant. (**G** and **H**) Rates of spontaneous or evoked burst firing for WT (black) or null (blue) PCs. Values in the parentheses represent numbers of cells that did or did not show bursting firing, as indicated. Differences between WT and null cells are significant (*P* = 0.0043, Fisher’s Exact Test, 2-tailed). (**I** and **J**) Percentages of WT (black) or null (blue) PCs that fired 3 or fewer APs versus more than 3 APs. Values in parentheses represent numbers of cells that fired fewer or more than 3 APs, respectively. Differences between genotypes are significant (*P* = 0.0101, Fisher’s Exact Test, 2-tailed).

PCs are known to fire spontaneously, often with burst firing, and we observed that behavior here for both genotypes (Figure 3, A–D; WT: black, null: blue). The percentage of PCs showing spontaneous firing was similar for WT cells (84%) and null (76%) PCs (Figure 3, E and F, no significant differences between genotypes). In contrast, a greater percentage of null PCs (42%) showed this burst firing pattern compared with WT (16%) (Figure 3, G and H, *P* = 0.0043). We separated PCs into 2 groups, those that fired only tonically and those that also showed burst firing, and then recalculated AP firing frequencies for both genotypes. Even after this separation, the AP firing frequency of null PCs remained significantly lower than WT (Supplemental Figure 2). Finally, while 20% of WT PCs fired 0 APs or fired 3 or fewer repetitive APs in response to depolarizing current injections, a greater percentage of null PCs (40%) showed this behavior. The difference was significant between genotypes (Figure 3, I and J, *P* = 0.0101). These groups of nonfiring or low-firing cells were not included in input-output analyses.

Quantification of passive and active membrane electrical properties for WT and null PCs is shown in Supplemental Table 2 and Supplemental Figure 3. Values for input resistance (panel A), minimum current required for evoking APs (panel C), and AP half-width (panel D) were increased in null PCs compared with WT. Values for maximal AP rise rate (panel E), maximal AP decay rate (panel F), and total APs fired (panel H) were decreased in null PCs compared with WT. There were no significant differences in resting membrane potential (panel B) or peak AP amplitude (panel G) between genotypes. Taken together, these results suggest that null PCs have reduced levels of functional ion channels resulting in reduced excitability.

To examine whether hyperpolarization-activated cyclic nucleotide-gated (HCN) channel activity contributed to the observed differences in spontaneous and bursting activities of *Scn1b* null PCs compared with WT, we measured HCN channel–mediated potential changes expressed as voltage sags in response to –60 pA hyperpolarizing current injection. No significant differences in sag amplitude or area were observed between genotypes (Supplemental Figure 4).

### Scn1b null PCs have reduced transient and resurgent I_Na_.

**Figure 4:**
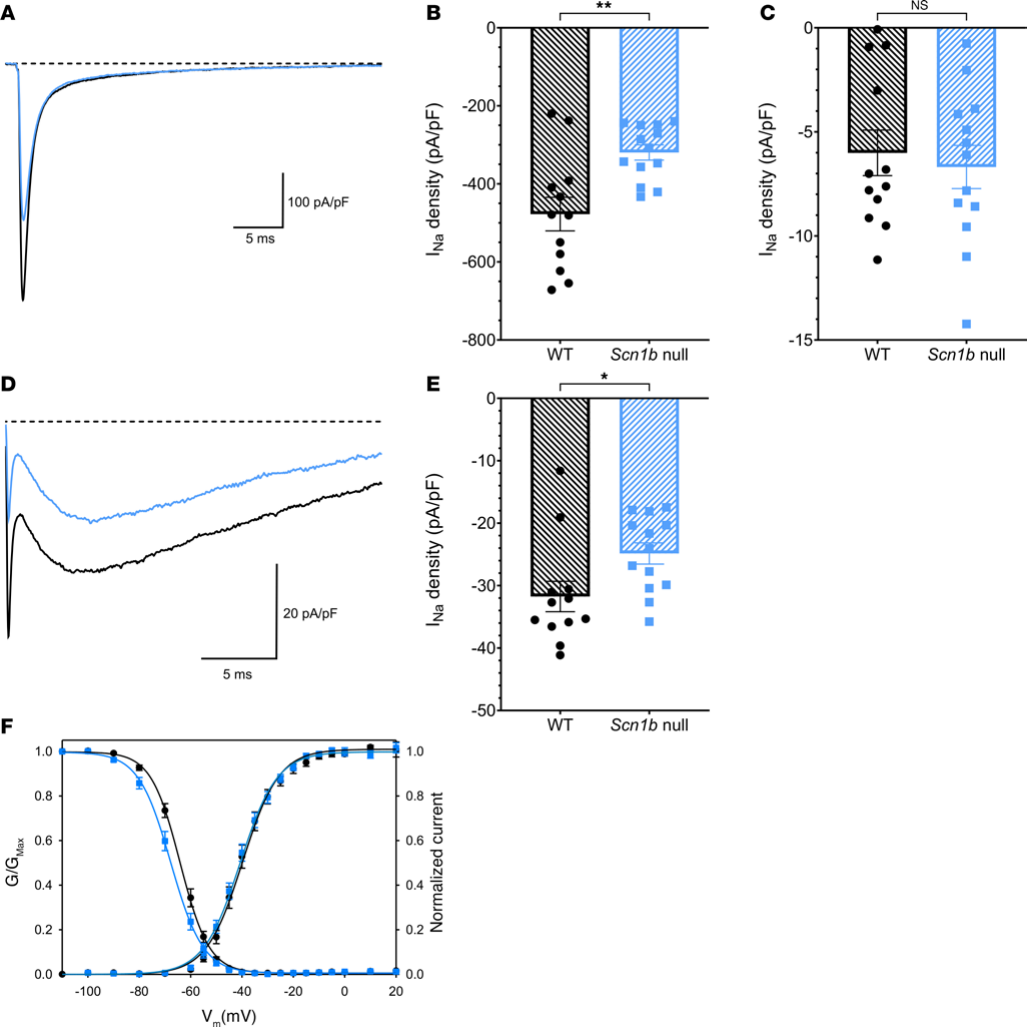
*Scn1b^–/–^* PCs have reduced transient and resurgent I_Na_ densities. (**A**) Representative transient I_Na_ traces from WT (black) or null (blue) PCs. Currents were evoked by a depolarizing pulse from –120 to –30 mV. Transient I_Na_ was measured at the peak, and persistent I_Na_ was assessed as the average current between 48 and 50 ms. (**B**) Maximal transient I_Na_ density. (**C**) Persistent I_Na_ density. (**D**) Representative resurgent I_Na_ density traces recorded in response to a repolarizing pulse to –40 mV, following a prepulse to +30 mV. A fast tail current is observed initially, followed by slow activating and inactivating resurgent I_Na_. (**E**) Resurgent I_Na_ density. (**F**) Activation and inactivation curves. No significant differences were observed between genotypes. Values are provided in Supplemental Table 3. Gmax, maximum conductance; Vm, membrane potential. Data are presented as means ± SEM for *N* = 12 WT or 13 null mice. **P < 0.005, *P < 0.05 (unpaired *t* test, 2-tailed *P* value).

We recorded whole-cell transient, persistent, and resurgent I_Na_ densities in acutely dissociated WT and null PCs to test the hypothesis that loss of β1 subunits results in lower I_Na_ via reduced chaperoning of VGSC α subunits to the plasma membrane. Figure 4A shows representative transient I_Na_ density traces from WT (black) and null (blue) PCs. Data are quantified in Figure 4B, showing a significant reduction in nulls compared with WT. Persistent I_Na_ density was not different between genotypes (Figure 4C). Representative resurgent I_Na_ density traces are shown in Figure 4D, and the data are quantified in Figure 4E. Resurgent I_Na_ density was significantly reduced in null PCs. We compared the voltage dependence of I_Na_ activation and inactivation in Figure 4F and found no differences between genotypes. These data are summarized in Supplemental Table 3.

### Scn1b null interneurons are hypoexcitable.

**Figure 5:**
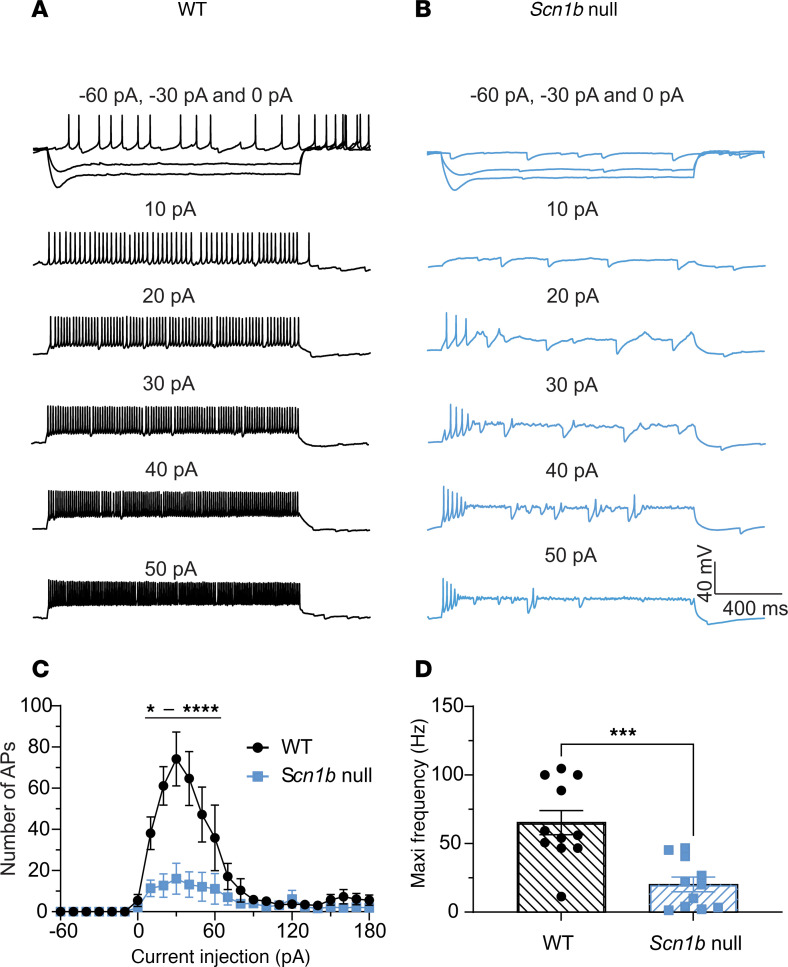
*Scn1b* null MLIs have increased AP initiation threshold and reduced firing frequency. Representative traces showing evoked repetitive firing of MLIs in lobule IV/V in sagittal cerebellar slices from WT (**A**) or null (**B**) mice. Repetitive AP firing was evoked by injections of 1,500 ms pulse currents of –60 pA to +180 pA (only selected –60 pA– to +50 pA–evoked responses are shown). Stronger depolarizing current injections were required to evoke repetitive firing in MLIs from null mice. (**C**) Null MLIs (blue) show reduced AP firing frequencies at all stimulation intensities compared with WT (black).* ^–^ **** represent *P* values from <0.05 to <0.0001. (**D**) Null MLIs show decreased maximal firing frequencies. *n* = 11 cells from 8 WT mice (black), and *n* = 11 cells from 9 null mice (blue). ***P = 0.0004, (2-way ANOVA for **C**, unpaired *t* test for **D**, 2-tailed *P* value).

We compared the AP firing rates of WT and null molecular layer interneurons (MLIs) using similar recording conditions as for PCs (Figure 5). This neuronal population includes 2 types of fast-spiking interneurons that innervate PCs, stellate cells and basket cells. Similar to our PC results, *Scn1b* null MLIs were hypoexcitable compared with WT (Figure 5, A and B, WT: black, null: blue), with fewer APs fired across the range of injected depolarizing currents (Figure 5C) and decreased maximal firing frequency (Figure 5D).

### Spontaneous GABAergic input to PCs is reduced in Scn1b null cerebellum.

**Figure 6:**
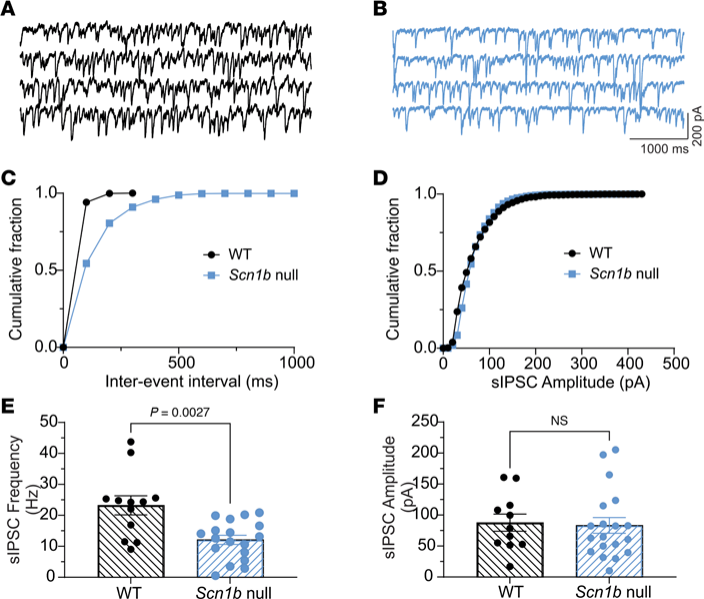
*Scn1b* null PCs have reduced sIPSC frequency but not amplitude. sIPSCs in PCs were recorded from a holding potential of –70 mV with a CsCl-based internal solution in the presence of 10 μM CNQX and 100 μM APV. (**A** and **B**) Representative traces showing sIPSCs recorded from P14–20 WT (black) or null (blue) PCs. (**C** and **D**) Comparisons of cumulative fraction of interevent intervals (**C**) or amplitudes (**D**) of sIPSCs from the same cells shown in **A** and **B**. (**E** and **F**) Comparisons of differences in mean frequencies (**E**) or amplitudes (*P* = 0.0027, Mann-Whitney test, 2-tailed) (**F**) of sIPSCs between WT and null mice. Recordings from 12 cells from 11 WT mice or 18 cells from 13 null mice.

Reduced MLI excitability is expected to decrease GABA release from presynaptic terminals onto PCs. We compared spontaneous inhibitory postsynaptic currents (sIPSCs), in the presence of 6-Cyano-7-nitroquinoxaline-2,3-dione (CNQX) and amino-5-phosphonopentanoic acid (APV) to block glutamatergic responses, in WT and *Scn1b* null PCs to investigate changes in GABAergic synaptic transmission onto these cells. Figure 6, A and B, show representative sIPSCs from WT (black) and null (blue) PCs, respectively, recorded under conditions of symmetric [Cl^–^] inside and outside using a CsCl-based internal solution and artificial cerebrospinal fluid (ACSF) in the external solution, resulting in inward GABA_A_ receptor-mediated currents at a holding potential of –70 mV (25). We observed a reduced cumulative fraction of interevent intervals, or frequency (Figure 6C), but not amplitude (Figure 6D) of sIPSC events in null compared with WT PCs, indicating reduced GABA release from presynaptic MLIs. Comparisons of mean sIPSC frequencies and amplitudes between WT and null PCs are shown in Figure 6, E and F.

### Spontaneous glutamatergic input to Scn1b null PCs is increased.

**Figure 7:**
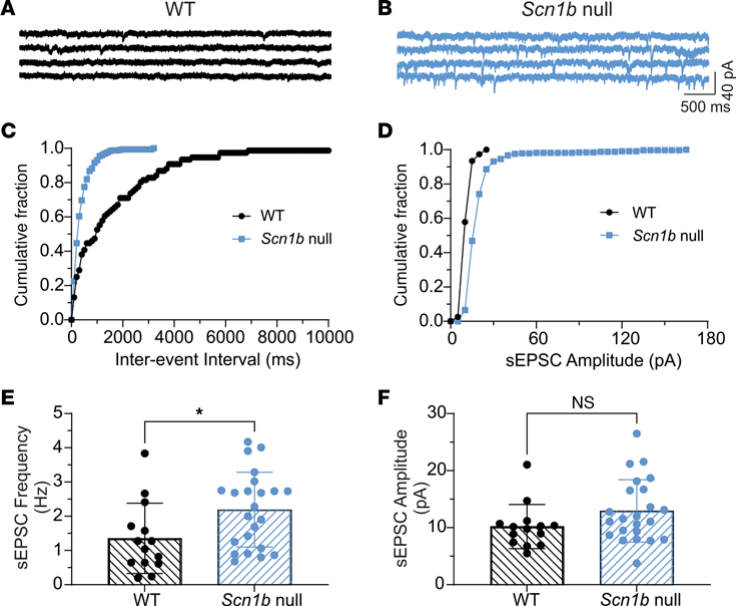
*Scn1b* null PCs have increased sEPSC frequency but not amplitude. sEPSCs in PCs were recorded from a holding potential of –70 mV in the presence of 10 μM bicuculline. (**A** and **B**) Representative traces showing sEPSCs recorded from P14–20 WT (black) or null (blue) PCs. (**C** and **D**) Comparison of cumulative fraction of interevent intervals (**C**) or amplitudes (**D**) of sIPSCs from the same cells shown in **A** and **B**. (**E** and **F**) Comparisons of differences in mean frequencies (**E**) or amplitudes (**F**) of sEPSCs between WT and null mice. Recordings from 14 cells of 8 WT mice or 23 cells of 16 null mice. *P = 0.026 (unpaired *t* test, 2-tailed).

Decreased GABAergic synaptic transmission to PCs may result in increased glutamatergic synaptic responses, though feedback loops between PC neurons and their excitatory inputs may make the situation more complex. To test this hypothesis, we recorded PC spontaneous excitatory postsynaptic currents (sEPSCs) in WT and null cerebellar slices from a holding potential of −70 mV in the presence of bicuculline to block GABAergic responses (Figure 7). Representative traces showing sEPSCs recorded from P14–20 WT or *Scn1b* null PCs are shown in Figure 7, A and B (WT: black, null: blue). The sEPSC frequency (cumulative fraction of interevent intervals, Figure 7C), but not amplitude (Figure 7D), was increased in null compared with WT PCs, suggesting increased glutamate release from presynaptic climbing fibers (CFs) or PFs. Comparisons of mean sEPSC frequencies and amplitudes between WT and null mice are shown in Figure 7, E and F, respectively.

### Scn1b deletion alters short-term synaptic plasticity at CF-PC synapses.

**Figure 8:**
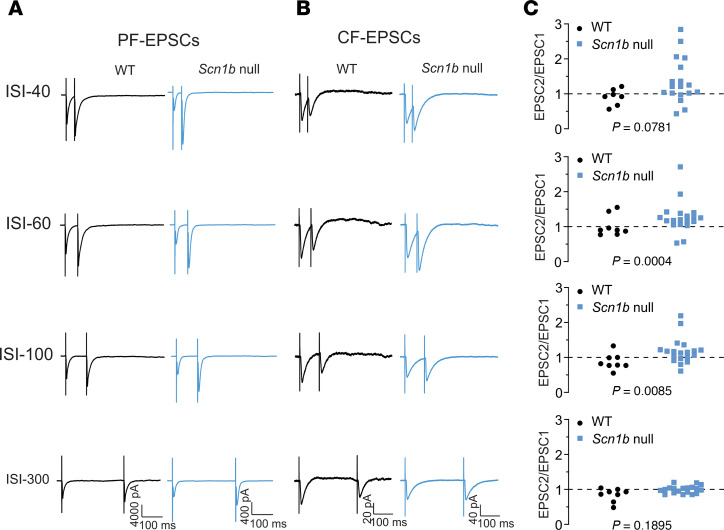
*Scn1b* deletion alters short-term synaptic plasticity at CF-PC synapses. Paired-pulse evoked EPSCs were recorded in PCs by stimulation of parallel fibers (PFs) in the pier of the external molecular layer or climbing fibers (CFs) in white matter at different interstimulus intervals (ISI 40, 60, 100, or 300 ms, as indicated). Amplitudes of EPSCs evoked by field stimulation varied from cell to cell because of individual cell variation and position of the stimulating electrode. WT cells (black) showed paired-pulse facilitation (PPF) at PF-PC synapses (**A**, left) and paired-pulse depression (PPD) at CF-PC synapses (**B**, left). *Scn1b* deletion (blue) promoted PPF at CF-PC synapses (**B**, right) with no significant effect on PF-PC synapses. Each trace is a representative example of 8 cells from 5 WT mice or 19 cells from 9 *Scn1b* null mice. (**C**) Statistical analyses of differences in short-term synaptic plasticity of CF-PC synapses at each indicated ISI between genotypes. EPSC2/EPSC1 ratios > 1 (above the dotted lines) were defined as PPF. EPSC2/EPSC1 ratios < 1 (below the dotted line) were defined as PPD. EPSC2/EPSC1 ratios = 1 (overlapping the dotted line) were defined as no interaction. CF-EPSCs evoked at ISI 60 showed significant changes in paired-pulse stimulation-evoked responses (*P* < 0.05, Fisher’s Exact Test, 2-tailed).

Alterations in sEPSC frequency suggest aberrant presynaptic mechanisms. To test this hypothesis, we investigated excitatory inputs to PCs from PFs (Figure 8A) and CFs (Figure 8B), respectively, by determining paired-pulse ratios of PF-EPSCs and CF-EPSCs recorded in current clamp mode from P14–20 WT and null cerebellar slices (WT: black, null: blue). Consistent with previous work (26–28), paired-pulse stimulation of PFs in WT slices evoked PPF, while paired-pulse stimulation of CFs evoked PPD. *Scn1b* deletion did not affect paired-pulse responses of PF-EPSCs but promoted PPF of CF-EPSCs: ISI 60 values were significantly increased in null vs. WT (*P* = 0.0004), while genotypic differences in ISI 40 (*P* = 0.0781) and ISI 100 (*P* = 0.00885) approached significance, suggesting preferential effects on transmitter release probability of CF terminals (Figure 8C).

### VGSC α and β subunit mRNA abundance.

VGSCs are responsible for AP initiation in all mammalian neurons and underlie the spontaneous firing observed in cerebellar PCs (29, 30). We performed a series of reverse transcription quantitative polymerase chain reaction (RT-qPCR) experiments to quantify VGSC α and β subunit mRNA abundance in *Scn1b* WT and null cerebellum at P15–17. No significant changes in the abundance of *Scn1a*, *Scn2a*, *Scn8a*, *Scn9a*, or *Scn4b* were detected between genotypes (Supplemental Figure 5, A, B, and E–G; WT: black, null: blue). In contrast, the mRNA abundance of *Scn3a* and *Scn4a* was significantly higher in null cerebellum compared with WT (Supplemental Figure 5, C and D; WT: black, null: blue). Increased levels of mRNA encoding the embryonic VGSC α subunit Nav1.3 may reflect the developmental delay observed in *Scn1b* null mice (10). However, the vanishingly low expression levels of *Scn4a* in the cerebellar samples, as detected by RT-qPCR with cycle threshold (Ct) values between 30 and 35, may indicate that this difference does not have biological significance.

### Scn8a protein and mRNA expression are unchanged in Scn1b null cerebellum.

Nav1.6 has been shown to predominantly underlie transient, persistent, and resurgent I_Na_ as well as repetitive firing in PCs (reviewed in ref. 31). We reported previously that the proportion of AISs expressing Nav1.6 was reduced in *Scn1b* null PCs analyzed by immunohistochemistry (IHC) in brain slices (20). We reexamined that result here in light of new techniques and reagents to better visualize anti-VGSC antibody staining in the brain (32). We quantified the expression of Nav1.2 and Nav1.6 at the PC AIS in both WT and null P17 cerebellar slices that had been fixed using glyoxal instead of paraformaldehyde, a technique that results in higher quality immunostaining of ion channels at the AIS (32). We were not able to obtain high-quality anti-Nav1.1 IHC staining using currently available antibodies. In both WT and null cerebellum, we observed concentrated Nav1.6 staining at the PC AIS (Supplemental Figure 6A). In contrast, Nav1.2 staining was undetectable at the PC AIS for both genotypes at this time point (Supplemental Figure 6B). We quantified the proportion of AISs positive for Nav1.6 by staining with anti-Kv1.2, which selectively labels Pinceau synapses surrounding the PC AIS (33), as a marker. In contrast with our previous work, we found no significant difference in the proportion of Nav1.6-positive PC AISs between genotypes (97.24% ± 0.58% in WT, 97.10% ± 0.61% in *Scn1b* null mice, *n* = 27 fields of view). We attribute this modified result to the use of a higher quality anti-Nav1.6 antibody combined with glyoxal fixation.

To examine *Scn8a* expression in *Scn1b* null cerebellum in greater detail, we used RNAscope Fluorescent Multiplex assays to quantify levels of *Scn8a* mRNA expression between genotypes for PCs, MLIs, and PC layer interneurons (PLIs) using a combination of anti-calbindin antibody and probes for *Scn8a*, *Pvalb*, and *Nxph1* (Supplemental Figure 7). Analysis of wide-field (Supplemental Figure 7, A and B) and high-resolution images (Supplemental Figure 7, C, D, F, G, I, and J) showed that *Scn8a* levels in all 3 cerebellar cell types were similar between genotypes. Wide-field and high-resolution images of IHC-RNAscope staining using anti-calbindin antibody + *Pvalb* + *Scn8a* were similar for both WT (control) and null PCs (Supplemental Figure 7, A–C). The data are quantified in panel E. High-resolution images showing IHC-RNAscope staining for *Pvalb* *+* *Scn8a* showed comparable *Scn8a* signal levels in MLIs between genotypes (Supplemental Figure 7, F and G and quantified in panel H). Finally, high-resolution images showing IHC-RNAscope staining for *Nxph1* *+* *Scn8a* showed comparable *Scn8a* signal levels in PLIs between genotypes (Supplemental Figure 7, I and J and quantified in K).

### Tetrodotoxin-sensitive VGSC α subunit protein expression is unchanged between genotypes.

Equilibrium tritiated-saxitoxin (^3^H-STX) binding is a highly sensitive method to assess the expression of tetrodotoxin-sensitive (TTX-S) VGSC α subunit proteins in cells and tissues (34). Because STX binds to TTX-S VGSCs with a 1:1 stoichiometry, it can be used to directly quantify VGSC α subunit proteins. We used ^3^H-STX to quantify total (plasma membrane + intracellular) TTX-S VGSC expression in WT null cerebellar membrane preparations, as in our previous work (35, 36). Consistent with the RT-qPCR results presented above for mRNAs encoding TTX-S VGSCs, specific ^3^H-STX binding (fmol/mg protein) in the cerebellum was not different between genotypes, suggesting that levels of cerebellar TTX-S VGSC protein expression were not impacted by *Scn1b* deletion (Supplemental Figure 8; WT: black, null: blue). These results may not reflect the increased abundance of *Scn3a* in null cerebellum due to the lack of effects on expression of the major channels (*Scn1a* and *Scn8a*) in this brain region. Overall, this result suggests that our observation of reduced I_Na_ density in null PCs is due to reduced chaperoning of VGSC α subunits to the plasma membrane where they are functional.

### Scn1b null PC AISs have increased length.

AIS length alterations can occur in multiple neurological disorders, particularly in the context of altered excitability (37). Because of the reduced excitability observed in *Scn1b* null cerebellum, we measured PC AIS lengths in brain slices. PC AIS lengths were assessed in cerebellar lobules IV (Supplemental Figure 9, A and D), VI (Supplemental Figure 9, B and E), and VIII (Supplemental Figure 9, C and F) of both genotypes at P17, using anti-AnkG as a marker for the AIS and anti-calbindin antibody as a marker for PCs. We observed that AnkG-positive AIS lengths in all lobules were greater in *Scn1b* null neurons compared with WT, with differences in mean values of 1.99 ± 0.31 μm (lobule IV), 1.17 ± 0.37 μm (lobule VI), and 1.14 ± 0.37 μm (lobule VIII). The same length increase was observed in the combined lengths from all lobules, with an overall difference in means of 1.40 ± 0.20 μm (Supplemental Figure 9G).

### PC Sholl radius is reduced in Scn1b null mice.

PC excitability requires correct formation of elaborate dendritic arbors so that synaptic connections can form in specific locations. We used Sholl analysis to evaluate the branching of PC dendritic trees in P12–19 WT and *Scn1b* null brains (Supplemental Figure 10, A and C, number of mice/number of cells [*N*/*n*]: WT 11/24, null 8/27). Significant differences in the number of intersections of the dendrites were observed between genotypes at approximately 100 mm from the soma, as indicated in Supplemental Figure 10A. Comparing the largest Sholl radius across neurons, we observed that WT PC dendritic arbors were larger than null (Supplemental Figure 10B; *N*/*n*: WT 11/24, null 8/27, *P* < 0.005), which may reflect the overall developmental delay in the *Scn1b* null mouse phenotype.

## Discussion

Ataxia is a hyperkinetic movement disorder that includes reduced coordination and balance, clumsy and irregular movements, dysarthria or difficulty speaking, kinetic tremor, and a wide-based stance (38). Early-onset cerebellar ataxias have been linked to genetic disorders (39), including the DEEs with evidence for altered gaits in both patients and mouse models (40–42). Here we present evidence that *Scn1b* null mice, which model DS and DEE52, are ataxic with a slow and irregular gate and a wide-based stance. Interestingly, connections between cerebellar deficits and cognitive decline in DS have also been suggested (43, 44), possibly implicating the cerebellum in more than ataxia in DEE.

*SCN1B* is essential for life. Pathogenic variants in *SCN1B* can impact multiple organ systems, including brain and heart (9, 45–47). β1 subunits are multifunctional: As sodium and potassium channel modulators and chaperones, they make critical contributions to the regulation of cellular and network excitability (36, 48–54) as well as maturation of GABAergic signaling (24). As CAMs, they participate in brain and heart development (9, 46, 55) and contribute to transcriptional regulation as substrates for regulated intramembrane proteolysis (52, 56–58). *Scn1b* null mice have spontaneous generalized seizures, increased sensitivity to hyperthermia-induced seizures, ataxia, failure to thrive, cardiac arrhythmia, and SUDEP (10, 24, 58, 59). Excitability changes in *Scn1b* null mice are cell type specific (10, 22, 60, 61). Finally, I_Na_ density heterogeneity between adjacent excitatory neurons, which normally regulates spike pattern diversity and network synchronization in the cortex, is impaired in *Scn1b* null mice, suggesting a mechanism for epileptogenesis (62).

*Scn1b* null mice have abnormal cerebellar development. We showed that VGSC β1 subunits normally function in *trans* as IgSF-CAMs to drive neurite outgrowth in mouse CGNs (19). *Scn1b* null mouse cerebellar PFs have defective fasciculation and axon pathfinding in vivo, with CGNs migrating aberrantly into the inner granule cell layer (20). Cultured *Scn1b* null CGNs have reduced resurgent I_Na_ (20). In addition, *Scn1b* null mice have defective axon pathfinding and fasciculation of the corticospinal tract, a structure that also contributes to motor coordination (64). Interestingly, the null mouse model of *Cntn1*, encoding the IgSF-CAM contactin, which is a functional binding partner of VGSC β1 subunits (65, 66), has a similar phenotype to *Scn1b* null mice, including death by P18, severe ataxia, and cerebellar micro-organization defects with aberrant CGN axon guidance (67).

Here, we show that VGSC β1 protein expression in WT mouse cerebellum, assessed using a CRISPR-derived mouse model containing an in-frame V5 epitope tag at the β1 C-terminus, is widespread, with high levels of immunostaining in the ML, in PC AISs, and in the granule cell layer. Similar to our previous work in *Scn1b* null cortical neurons, we show here that the intrinsic excitability of *Scn1b* null PCs and MLIs is remarkably reduced compared with WT. The increased AIS length observed in null PCs may indicate an adaptive response of these cells to increase their intrinsic excitability. The frequency of sIPSCs measured in PCs is also reduced, reflecting the reduced intrinsic excitability of MLIs. In contrast, the frequency but not amplitude of sEPSCs measured in PCs is increased, suggesting increased presynaptic release of glutamate because of reduced firing of inhibitory neurons. Preferential promotion of PPF at CF-EPSCs also supports a presynaptic mechanism. However, this complex result may also reflect the highly disordered state of PF migration and subsequent formation of synaptic connections in the null cerebellum (20).

*Scn1b* null mouse cortical fast-spiking interneurons and layer 6 pyramidal neurons have decreased transient I_Na_ density, reflecting the essential role of VGSC β1 subunits in chaperoning VGSC α subunits to the plasma membrane (60). In addition, acutely isolated *Scn1b* null PCs show a hyperpolarizing shift in the voltage dependence of I_Na_ inactivation, predicting reduced channel availability compared with WT (68). Here we show that transient and resurgent I_Na_ densities are reduced in PCs, consistent with our findings of increased input resistance and decreased values for maximal evoked AP firing rate, maximal AP rise rate, and maximal AP decay rate, in null PCs compared with WT. These results are similar to those reported for PCs in *Scn1a^+/–^* mice, which also model DS with ataxia (41). Overall, reduced inhibitory PC output to the deep cerebellar nuclei is consistent with the ataxic phenotype of *Scn1b* null mice. Our data showing neuronal hypoexcitability in the face of largely unchanged values for VGSC α subunit mRNA abundance (except for *Scn3a*, which may reflect the developmental delay observed in *Scn1b* null brains) and protein subcellular localization predict that the major mechanistic deficit in *Scn1b* null cerebellum, in addition to disrupted neuronal pathfinding and fasciculation, is reduced chaperoning of VGSC α subunit proteins to the plasma membrane in the absence of VGSC β1 subunits, resulting in reduced I_Na_ density and reduced excitability (36).

Emerging evidence shows that, in addition to motor control, the cerebellum can influence seizure networks (14, 69). Both inhibition and excitation of PCs have been shown to be effective in attenuating seizures in a mouse model of temporal lobe epilepsy, demonstrating the ability of the cerebellum to variably modulate the excitability of other brain regions (70). Importantly, excitation of deep cerebellar nuclei reliably decreased the frequency of generalized spike and wave discharges in a mouse model of absence epilepsy (71). The specificity of neuronal subtypes stimulated appears to be critical. Closed-loop optogenetic stimulation of a subpopulation of excitatory glutamatergic deep cerebellar fastigial neurons was more effective in attenuation of mouse hippocampal seizures compared with broad excitation of fastigial neurons as a whole (72). This body of work has led to proposals for therapeutic strategies for seizure attenuation based on neuronal cell type–specific modulation in the cerebellum. Thus, while deficits in *Scn1b* null brain are not limited to the cerebellum (60), increased output from excitatory projections from the deep cerebellar nuclei may be effective in inhibiting seizures. In the *Scn1b* null mouse model, decreased cerebellar output may contribute to the severe seizure phenotype (10, 11) because of the inability of the cerebellum to provide a “seizure brake.” Taken together, our work suggests that cerebellar hypoexcitability may contribute to ataxia and seizure severity in DEE52.

## Methods

### Sex as a biological variable.

Approximately equal numbers of male and female pups were used, prior to weaning, in all experiments. Sex as a biological variable was not considered. Findings are expected to apply to both sexes.

### Animals.

All animals were housed at the University of Michigan in the Unit for Laboratory Animal Medicine.

WT (*Scn1b^+/+^*) and *Scn1b* null (*Scn1b^–/–^*) mice were bred from *Scn1b^+/–^* mice congenic on the C57BL/6J background for more than 20 N generations (10). Male and female pups were used prior to weaning in all experiments.

The β1-V5 mice, containing an in-frame V5 epitope tag at the β1 C-terminus, were generated using CRISPR/Cas9 gene-editing technology. Exon 5 of Ensembl gene model Transcript *Scn1b*-001 (ENSMUSE00000533876) encodes the β1 subunit C-terminus. The CRISPOR algorithm (73) was used to identify specific guide RNA (gRNA) targets. gRNAs predicted to cut the chromosome near codon 218 in exon 5 were subcloned in plasmid pX330-U6-Chimeric_BB-CBh-hSpCas9v (74) and tested for chromosome breaks. pX330 plasmids were coelectroporated with a puromycin selection plasmid (sourced in-house) into mouse embryonic stem cells. After drug selection, surviving cells were pooled and genomic DNA was prepared. PCR with primers (5′-GGGACTCTAGTCTATCCCATTCTATTC-3′ forward, 5′-CTATGTGCAAATCAGCAAAAAGAAG-3′ reverse) spanning the predicted cut site were used to generate amplicons for Sanger sequencing. The process was similar to that described previously (75, 76). Sequence chromatograms of amplicons were evaluated to determine if small insertions/deletions caused by nonhomologous endjoining repair of chromosome breaks were present. Using this method, gRNA C60G3, targeting the sequence 5′-CTGGCGATGCGCAGACCCGT-3′ (protospacer adjacent motif = CGG) 3′ with a high specificity score (cutting frequency determination score of 96; ref. 77), was found to be the most active. The sequence of the single-stranded oligonucleotide DNA donor was as follows: CCTTCCCTTGCACAGCTCAGAATACCTGGCCAT TACATCCGAGAGCAAAGAAAACTGTACAGGCGTCCAGGTGGCTGAA **GGCAAGCCTATCCCTAACCCTCTCCTCGGCCTC GATTCTACC**tagCGCTGGTAAGGTTGATGGAAAGACGGGATCCTGCGCATCGCCAGCCAGGAGGGCTCAGGGACACACTCTTAAGTCT, in which exon 5 is underlined, the V5 coding sequence is shown in bold, and lowercase letters indicate the termination codon. The use of high-specificity sgRNA and ESPCAS9 has been shown to make off-target hits in mouse models unlikely (78). After determining that C60G3 caused chromosome breaks, 5 ng/mL of pX330 plasmid was combined with 10 ng/mL of a single-stranded oligonucleotide DNA donor (https://www.idtdna.com/page).

CRISPR reagents were microinjected into fertilized mouse eggs produced by mating superovulated C57BL/6J female mice (Jackson Laboratory stock no. 000664) to C57BL/6J male mice as described (79). Three generation zero founder (G0) pups (#103, 131, and 167) were identified by Sanger sequencing of amplicons spanning *Scn1b* exon 5 and the V5 epitope tag. Each G0 founder was mated with WT C57BL/6J mice. Germline transmission was achieved with 1 founder, #167. This line was then backcrossed to C57BL/6J for 10 generations. The resulting homozygous *Scn1b^V5/V5^* knockin mice, called “β1-V5” throughout the paper, breed normally, have a normal neurological phenotype, and live normal lifespans.

### Western blot analysis of β1-V5 mouse brain.

Western blot analysis of mouse brain membranes was performed as previously described (8) using anti-β1_intra_ antibody (Cell Signaling Technology 13950, 1:1,000 dilution) and anti-V5 antibody (Invitrogen 46-0705, 1:1,000 dilution).

### Gait analysis.

P16 *Scn1b* WT and null littermate mice were subjected to footprint analysis (23). Paws were painted with nontoxic, washable paint, with forepaws painted red and hind paws painted blue. Gait was observed in a custom-made acrylic walkway with clear sides and bottom and a dark opaque cap at one end, with the walking area approximately 2′′ wide. Mice were acclimated to the walkway prior to the experiment. The bottom of the walkway was covered with a strip of filter paper. Mice were placed at one end of the walkway and allowed to walk freely to the other end until a series of at least 3 clear painted footprints along the paper was obtained. Forepaw and hind paw prints were identified and lines drawn between similar locations in each print. Stride lengths, stride widths, and step angles were measured separately for hind paws and forepaws. Statistical analysis (unpaired *t* test, 2-tailed) was performed using GraphPad Prism 10, with data presented as mean ± SEM. Gait videos and single-frame images were exported using Adobe Premiere Pro 2023.

### Electrophysiology — brain slice recordings.

Acute cerebellar slices were prepared as described (22). In brief, the brain was rapidly removed following euthanasia by isoflurane inhalation and decapitation. Both parasagittal (~200 μm) and coronal (transverse, ~250 μm) cerebellar slices were prepared from P14–20 WT or *Scn1b* null mice in ice-cold, oxygenated “slicing” solution saturated with 95% O_2_ /5% CO_2_. The slicing solution contained (in mM) 110 sucrose; 62.5 NaCl; 2.5 KCl; 6 MgCl_2_; 1.25 KH_2_PO_4_; 26 NaHCO_3_; 0.5 CaCl_2_; and 20 d-glucose (pH 7.35–7.4 when saturated with 95% O_2_ /5% CO_2_ at room temperature of 22**°**C–25**°**C). Slices were incubated in slicing solution for 15–20 minutes at room temperature and then in a 1:1 mixture of slicing solution and ACSF, containing (in mM) 125 NaCl; 2.5 KCl; 1 MgCl_2_; 1.25 KH_2_PO_4_; 26 NaHCO_3_; 2 CaCl_2_; and 20 d-glucose (pH 7.35–7.4) in a holding chamber aerated continuously with 95% O_2_ /5% CO_2_ at 35**°**C for 30 minutes and finally being transferred to ACSF for at least another 30 minutes before use. The coronal cerebellar slices were used primarily for recordings of paired-pulse stimulation of PF-EPSCs because the coronal or transverse slices best preserve the PFs, whereas thin parasagittal slices cut off most PFs and can be used for recordings of paired-pulse stimulation CF-EPSCs with minimized involvement of activations of PF-mediated responses. Since parasagittal cerebellar slices can be easily used to identify lobule structures, they were used for all other electrophysiological and IHC experiments.

For electrophysiological recording, each cerebellar slice was transferred to a recording chamber, where it was perfused (2–3 mL/min) with ACSF bubbled continuously with 95% O_2_ /5% CO_2_. PCs or MLIs in lobules IV/V or VI in parasagittal cerebellar slices were visually identified based on their size, shape, and location using a Nikon E600FN upright microscope equipped with Nomarski optics (40× water immersion objective). For recording of AP firing, recording electrodes had a resistance of 3–6 MΩ when filled with a K-gluconate–based pipette solution consisting of (in mM) 140, K-gluconate; 4, NaCl; 0.5, CaCl_2_; 10, HEPES; 5, EGTA, 5 phosphocreatine; 2, Mg-ATP; and 0.4, GTP (pH 7.2–7.3 adjusted with KOH). Repetitive firing pattern and frequency of APs of individual neurons were examined using the whole-cell current clamp recording technique. Repetitive AP firing was evoked by injections of a series of 1,500 ms depolarizing currents varying from –60 pA to 180 pA at 10 pA step from their resting membrane potentials. The threshold for detection of APs was set at 0 mV, and thus only spikes that reached 0 mV or overshot 0 mV were counted as APs. Input-output curves of repetitive AP firing were constructed by plotting the number of APs evoked against the currents injected. Because some cells could not fire any APs or fired no more than 3 APs, these cells were not included in input-output analysis. HCN channel–mediated potential changes were examined at the voltage sag evoked by –60 pA current injection. Voltage sag amplitudes or HCN potentials were measured as the maximum membrane hyperpolarization minus the steady-state potential just prior to offset of the –60 pA current injection pulse as indicated by the arrow in Supplemental Figure 1C. For recording spontaneous synaptic responses in PCs, whole-cell voltage-clamp recording techniques were used with 2 pipette solutions. For recording sIPSCs, the pipette solution consisted of (in mM) 140, CsCl; 4, NaCl; 0.5, CaCl_2_; 10, HEPES; 5, EGTA, 5 phosphocreatine; 2, Mg-ATP; and 0.4, GTP (pH 7.3 adjusted with CsOH). sIPSCs were recorded at a holding potential of –70 mV in the presence of CNQX (10 μM) and APV (50–100 μM) in the external solution to block glutamate receptor–mediated synaptic responses. For recordings of miniature IPSCs (mIPSCs), 0.5 μM TTX was added subsequently to the external solution in addition to CNQX and APV to block spontaneous firing-evoked release of neurotransmitters. For recording sEPSCs, the pipette solution consisted of (in mM) 140, K-gluconate; 4, NaCl; 0.5, CaCl_2_; 10, HEPES; 5, EGTA, 2, Mg-ATP; and 0.4, GTP (pH 7.3 adjusted with KOH). sEPSCs were recorded at a holding potential of –70 mV in the presence of bicuculline (10 μM) in the external solution to block GABA_A_ receptor-mediated synaptic responses. Similarly, miniature EPSCs (mEPSCs) were recorded in the presence of 0.5 μM TTX in the external solution in addition to bicuculline. For recordings of PF-EPSCs, a monopolar 2 MΩ tungsten stimulating electrode (FHC) was placed on the pier of the external ML of a transverse cerebellar slice while a whole-cell patch clamp recording electrode was patched on a downstream PC. For recordings of CF-EPSCs, the same stimulating electrode was placed in the white matter, occasionally in the granule cell layer, of a parasagittal cerebellar slice. Isolated stimuli were generated from a Grass S88 stimulator at a frequency of 0.2 Hz, 0.1 ms duration, varied voltages that caused 50%–60% of maximum amplitude of responses and different ISIs (40 ms, 60 ms, 100 ms, and 300 ms).

All electrophysiological signals were amplified with a Multiclamp 700B amplifier (Molecular Devices), filtered at 2–4 kHz, and digitized at 20 kHz for offline analysis. Data were acquired with a Digidata 1440A interface and analyzed using pClamp11 off-line. All experiments were performed at room temperature of 22**°**C–25**°**C. Data were analyzed offline as described previously (22). For analysis of spontaneous synaptic transmission, spontaneous synaptic events were first screened automatically in a nonstop mode using MiniAnalysis 6.0 software (Synaptosoft Inc.) with a set of prespecified parameters, including detection thresholds for event amplitudes and areas, which effectively eliminated those unwanted noises. The detected events were then accepted or rejected manually with a new event amplitude detection threshold at 5 folds over the baseline for sIPSCs/mIPSCs and 3 folds over the baseline for sEPSCs/mEPSCs. A period of 10 ms baseline was averaged prior to each event and was used for calculating the event amplitude. The key to distinguish synaptic events from noises is that real synaptic events (sIPSCs or sEPSCs) have a fast-rising phase (usually < 1 ms) and slow decay phase, whereas noises do not have these features. The mean frequency and amplitude of sIPSCs or sEPSCs were calculated from recordings of a 2-minute period from each individual cell.

### Electrophysiology — recording of acutely dissociated PCs.

Parasagittal cerebellar slices were prepared as above but maintained in 100% slice solution at room temperature until dissociation. One slice was incubated at 35°C in oxygen-saturated HBSS supplemented with 10 mM HEPES with 1.5 mg/mL protease type XIV (Sigma) for 14 minutes. Tissue was washed 3 times with oxygen-saturated, ice-cold, low-calcium HBSS (1:10 HBSS with calcium and magnesium/HBSS calcium and magnesium free) containing 10 mM HEPES. HBSS was replaced with ice-cold, oxygen-saturated Na-isethionate solution (in mM; 140 Na-isethionate, 23 glucose, 15 HEPES, 2 KCl, 4 MgCl_2_, 0.1 CaCl_2_) and tritiated with fire-polished glass Pasteur pipettes to suspend cells. Cells were allowed to settle on a glass coverslip for 10 minutes prior to recording. All recordings were acquired within 1.5 hours of dissociation. PCs were identified by their pear shape. Voltage clamp recordings were performed in the standard whole-cell configuration with conditions described before (80). Resurgent currents were recorded as described before (81). Cells were superfused with external VGSC recording solution containing in mM (30 NaCl, 1 BaCl_2_, 2 MgCl_2_, 45 CsCl, 0.2 CdCl_2_, 1 CaCl_2_, 10 HEPES, 20 TEA-Cl, and 100 d-glucose, pH 7.35 with CsOH, osmolarity 300–305 mOsM). Fire-polished pipettes were filled with VGSC internal recording solution containing (in mM) 115 CsCl, 0.5 CaCl_2_, 5 EGTA:CsOH, 10 HEPES, 5 Na_2_ phosphocreatine, 20 TEA, 2 Mg-ATP, and 0.4 GTP, with pH adjusted to 7.2–7.3 with CsOH. The junction potential was calculated to be 2.7 mV with all reported voltages uncorrected.

Electrophysiological data were statistically analyzed using a 2-tailed *t* test or 2-way ANOVA. For all statistical analyses, data were first tested for normal distribution. In the case of a *t* test, if data failed the variance test, the non-parametric Mann-Whitney test was used. When comparing the rates or frequencies of spontaneous firing, bursting firing or firing less than 3 APs between WT and null PCs, Fisher’s Exact test was used or, in the case of synaptic puncta, repeated measures ANOVA using GraphPad Prism 10. Statistical significance was considered at *P* < 0.05. Data are reported as mean ± SEM.

### RT-qPCR.

RNA was isolated from the cerebellum of *Scn1b* null and WT mice (P15–17) using the QIAGEN RNeasy Plus Kit according to the manufacturer’s instructions. Tissue was homogenized with a Tissue-Tearor (BioSpec Products, Inc.) followed by lysis through a sterile, 18-gage hypodermic needle and vortexing. RNA samples were run on a NanoDrop One Spectrophotometer (Thermo Fisher Scientific) to ensure adequate concentration and purity, followed by being stored at –80°C. cDNA was generated from 1–1.5 mg of RNA using Reverse Transcriptase SuperScript III (RT SS III), random primers (Invitrogen), and deoxynucleotide triphosphates (dNTPs) (Invitrogen). RNA, oligo dT primers, and dNTPs were incubated at 65°C for 5 minutes. Salt buffers, 0.1 M DTT, RNase Out (Invitrogen), and RT SS III were added, and reactions were incubated at 25°C for 5 minutes, 50°C for 60 minutes, and 70°C for 15 minutes. Quantitative PCR was performed using SYBR Green (Applied Biosystems) and gene-specific primers (*Scn1a*, *Scn2a*, *Scn3a*, *Scn4a*, *Scn8a*, *Scn9a*, *Scn4b*, *Actb*; Integrated DNA Technologies) on a QuantStudio 7 Flex Real-Time PCR System (Applied Biosystems). Gene-specific measurements of each cDNA sample were run in triplicate, along with the endogenous control gene *Actb* used for normalization, and then compared with the average WT expression levels. The relative expression levels of each gene were quantified using the comparative threshold (2^–ΔΔCt^) method of quantification. Sample sizes for each gene were *n* = 8–9 per group. Data are presented as mean fold change in gene expression ± SEM. Statistical significance (*P* < 0.05) of comparisons between genotypes was determined using 2-tailed *t* test using GraphPad Prism 10.

### RNAscope Fluorescent Multiplex assay.

RNAscope Fluorescent Multiplex assays (ACD Biosystems) were performed on formalin-fixed, paraffin-embedded cerebellar sections according to the manufacturer’s instructions. To study *Scn8a* levels in PCs and MLIs, sections were stained with *Mm-Scn8a-C1* (ACD Biosystems 434191) + *Mm-Pvalb-C3* (ACD Biosystems 421931) followed by IHC with anti-calbindin 1:1,000 (CK, Novus Biologicals NBP2-50028). To address *Scn8a* levels in PLIs, sections were stained with *Mm-Scn8a-C1* (ACD Biosystems 434191) + *Mm-Nxph1-C4* (ACD Biosystems 463401). Three cerebellar hemisphere images were obtained from different sections for each sample from assembled 63× tiles obtained with a Leica Stellaris tauSTED microscope. For signal quantification, regions of interest for the PC layer or the ML were hand drawn with Adobe Illustrator over each tile composite and then added as an additional channel to the multichannel image to facilitate the identification of the cell of interest using ilastik (https://www.ilastik.org/). After cell identification and segmentation with ilastik, intensity quantification was performed with ratiometric intensity counting (RIC), a custom intensity quantification package running as a napari plug-in. Sum intensity values were acquired per cell of interest and plotted as the mean ± SEM. Statistics were performed using unpaired 2-tailed *t* test in GraphPad Prism 10.

### ^3^H-STX binding.

Cerebellar membranes were prepared from P16–18 WT and *Scn1b* null mice as described (36). Equilibrium binding of a saturating concentration (5 nM) of C-11–labeled ^3^H-STX (20 Ci/mmol, American Radiolabeled Chemicals Inc.) in the presence or absence of 10 μM unlabeled TTX (Alomone Labs) to assess nonspecific binding was measured at 4°C for at least 1 hour and terminated using a vacuum filtration assay (36). To quantify ^3^H-STX binding, counts per minute values obtained from liquid scintillation counting (Packard Tri-carb 1900TR) were corrected for specific binding by subtraction of nonspecific values and then converted to decays per minute before normalization to total protein concentration using the BCA protein assay (Pierce, Thermo Fisher Scientific). Statistical analysis (unpaired 2-tailed *t* test) was performed using GraphPad Prism 10.

### Immunofluorescence imaging and analysis.

WT, *Scn1b* null, and β1-V5 mice were deeply anesthetized with isoflurane, then transcardially perfused with PBS followed by 4% paraformaldehyde. *Scn1b* WT and null mice for experiments in Figure 1 were perfused at P19 with 3% glyoxal solution (3% glyoxal from Sigma-Aldrich, 0.75% acetic acid, 20% ethanol, pH 5.0, modified from ref. 32) as an alternative to paraformaldehyde fixation. Brains were dissected, postfixed overnight in 4% paraformaldehyde or 3% glyoxal solution, sequentially submerged overnight in 10% and 30% sucrose, flash-frozen in OCT, and stored at –80°C. We generated 20 μm coronal sections on a Leica CM1850 cryostat and stored at –20°C. Immunofluorescence labeling was performed as previously described (25). Briefly, slides were rehydrated in 0.05 M phosphate buffer (PB) and blocked for at least 2 hours in blocking buffer (10% normal goat serum and 0.3% Triton X-100 in 0.1 M PB). Slides were incubated with primary antibodies (Supplemental Table 4) in blocking buffer overnight at room temperature. The next day, slides were washed 3 times for 10 minutes each with 0.1 M PB, incubated with Alexa Fluor–conjugated secondary antibodies in blocking buffer for 2 hours, washed 3 times for 10 minutes in 0.1 M PB, and then mounted with ProLong Gold + DAPI and stored at 4°C until imaging. Fluorescent images were acquired on a Nikon A1R confocal system with a Nikon FN1 microscope using a 20× 0.75 NA objective and Nikon NIS-Elements AR software.

For AIS length measurement, AnkG-positive segments adjacent to PC soma and extending into the granule cell layer in P17 brain slices were identified, and length was determined using the line measurement function in NIH ImageJ.

For identification of Nav1.6 or Nav1.2 expression in AISs, AIS locations were first determined using immunolabeling of Kv1.2, which is a component of the immature Pinceau synapse surrounding PC AISs visible at P17. Each Kv1.2-positive structure was then evaluated for the presence or absence of Nav1.6 or Nav1.2 signal. This technique provided clear identification of PC AISs as an alternative to AnkG due to abundant AnkG signal frequently present in the granule cell layer.

Statistical analyses of all immunofluorescence experiments (unpaired *t* test, 2-tailed) were performed using GraphPad Prism 10. Data are presented as mean ± SEM. Figures were assembled in Adobe Photoshop 2023.

### Sholl analysis of PCs.

PCs in brain slices were filled with biocytin (Sigma-Aldrich), with most slices containing 1–3 filled neurons. Slices were washed 3 times for 5 minutes in PBS, incubated in Alexa Fluor 568–conjugated streptavidin (Thermo Fisher Scientific S11226A, dilution 1:500) overnight, washed 3 times for 10 minutes in PBS, and coverslipped using ProLong Gold (Invitrogen). Images of individual PCs were manually skeletonized. Neurons that could not be conclusively skeletonized because of issues, such as inability to clearly trace along individual dendrites, incorrect angle of the brain slice, or incomplete biocytin fill, were not used for analysis. Sholl analysis was performed on skeletonized images using the Sholl analysis plug-in for NIH ImageJ. Data were analyzed using GraphPad Prism 10 (unpaired 2-tailed *t* test). Results are presented as mean ± SEM.

### Statistics.

Specific statistical tests are described in each subsection of the Methods as well as in the figure legends. *P* < 0.05 was considered statistically significant.

### Study approval.

All procedures were approved by the University of Michigan Institutional Animal Care and Use Committee (approval number PRO00012319).

### Data availability.

Data are available in the Supporting Data Values file.

## Author contributions

JW and HO performed gait analyses. CC generated the β1-V5 transgenic mouse line. YY conducted all brain slice electrophysiology experiments and analyses. LFLS performed patch clamp analysis of acutely dissociated PCs. SB and SH performed and analyzed the RT-qPCR experiments. ND performed and analyzed the ^3^H-STX binding experiments. AL performed and analyzed the RNAscope Fluorescent Multiplex assays. HO performed confocal immunofluorescence staining and analysis, AIS length analysis, and Sholl analysis. YY, HO, and LLI wrote the manuscript. LLI designed and oversaw the project.

## Supplementary Material

Supplemental data

Supplemental video 1

Supplemental video 2

Supporting data values

## Version 1 09/09/2025

Electronic publication
