# Supplementary material for: Ataxia and cerebellar hypoexcitability in a mouse model of SCN1B-linked Dravet syndrome
Source: JCI Insight. 2025 Sep 9;10(17):e187606. doi: 10.1172/jci.insight.187606 (PMC12487675; doi:10.1172/jci.insight.187606)
Supplement: Supplemental data [file jciinsight-10-187606-s071.pdf]

Supplemental Materials

Ataxia and cerebellar hypoexcitability in a mouse model of *SCN1B*-linked Dravet syndrome

Yukun Yuan[1], Heather A. O'Malley[1], Jesse J. Winters[1], Alfonso Lavado[2], Nicholas S. Denomme[1#], Shreeya Bakshi[1], Samantha L. Hodges[1], Luis Lopez-Santiago[1], Chunling Chen[1], and Lori L. Isom[1*]

[1]Department of Pharmacology, University of Michigan Medical School, Ann Arbor, MI 48109-5632

[2]Center for Pediatric Neurological Disease Research, St. Jude Children Research Hospital, Memphis, TN 38105-3678

*Corresponding author:

Lori L. Isom, PhD

Department of Pharmacology

University of Michigan Medical School

Ann Arbor, MI 48109-5632

734-936-3050

lisom@umich.edu

#Present address: Department of Psychiatry and Behavioral Sciences, Stanford University, Palo Alto, CA 94305

Supplemental Figures and Figure Legends

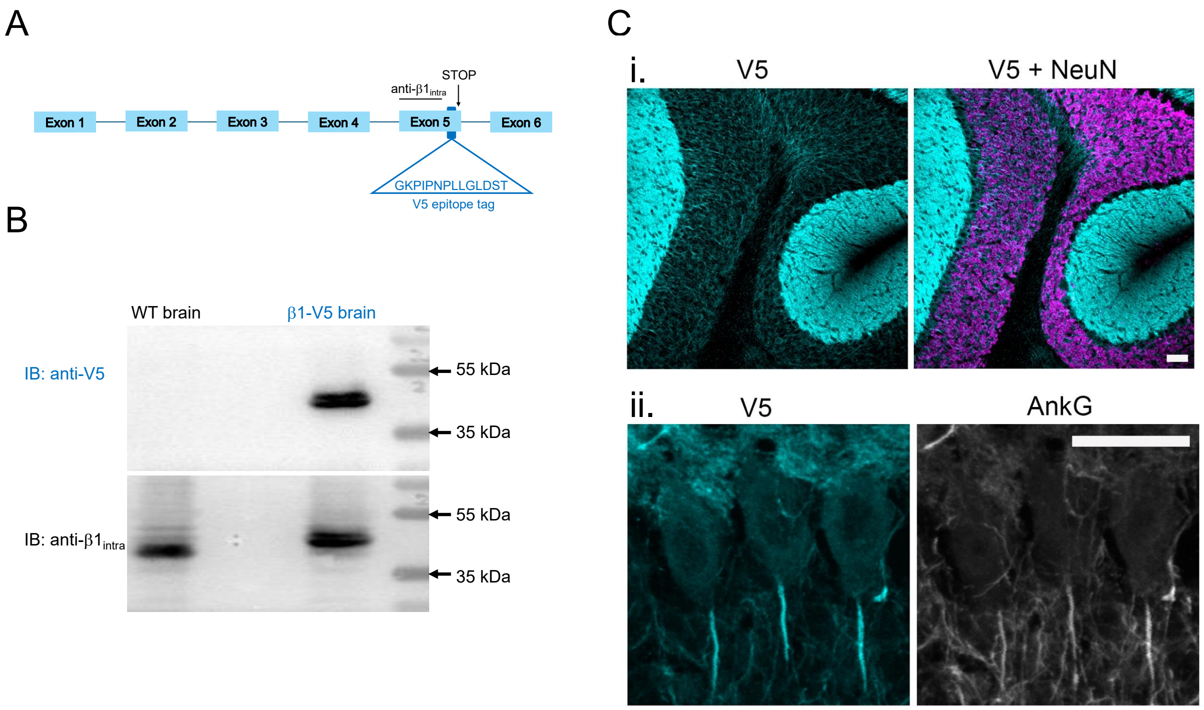

Supplemental Fig. 1. A and B. Generation of β1-V5 mice. A. Schematic showing the exon-intron structure of *Scn1b* and point of in-frame insertion into exon 5 prior to the termination codon (STOP). The location of the anti-$\beta1_{intra}$ epitope is indicated in exon 5. B**.** Western blot analysis of WT and *Scn1b*$^{V5/V5}$ mouse brain membranes with anti-V5 antibody showing anti-V5 signal in *Scn1b*$^{V5/V5}$ brain but not the WT brain (upper panel). The lower panel shows the same blot reprobed with anti-$\beta1_{intra}$ antibody, recognizes β1 protein in both strains. The MW of the β1-V5 protein is slightly larger that WT due to incorporation of the epitope tag. **C. β1 subunits are widely expressed in mouse cerebellum.** i: Expression of V5-tagged *Scn1b* protein in cerebellum of P19 β1-V5 mice. Cyan: V5, magenta: NeuN. ii: Specific expression of V5-tagged *Scn1b* protein at PC AISs positive for AnkG. Cyan: V5, grey: AnkG. Scale bars: 50 µm (panels A-Ca), 25 µm (panel Cb).

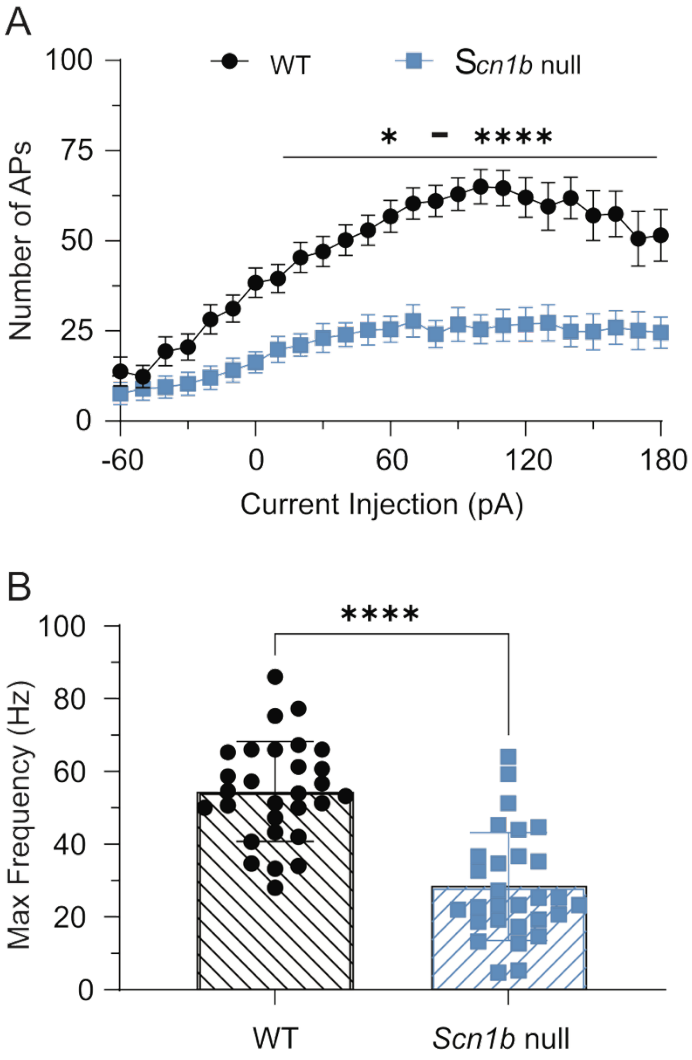

Supplemental Fig. 2. **Firing properties of PCs that did not show burst firing. A.** Input-output curves of AP firing for WT or *Scn1b* null PCs that did not show burst firing activity. **B.** Comparison of maximal firing frequency between genotypes. Values are mean ± SEM of 29 PCs from 20 WT mice or 35 PCs from 29 *Scn1b* null mice, respectively.

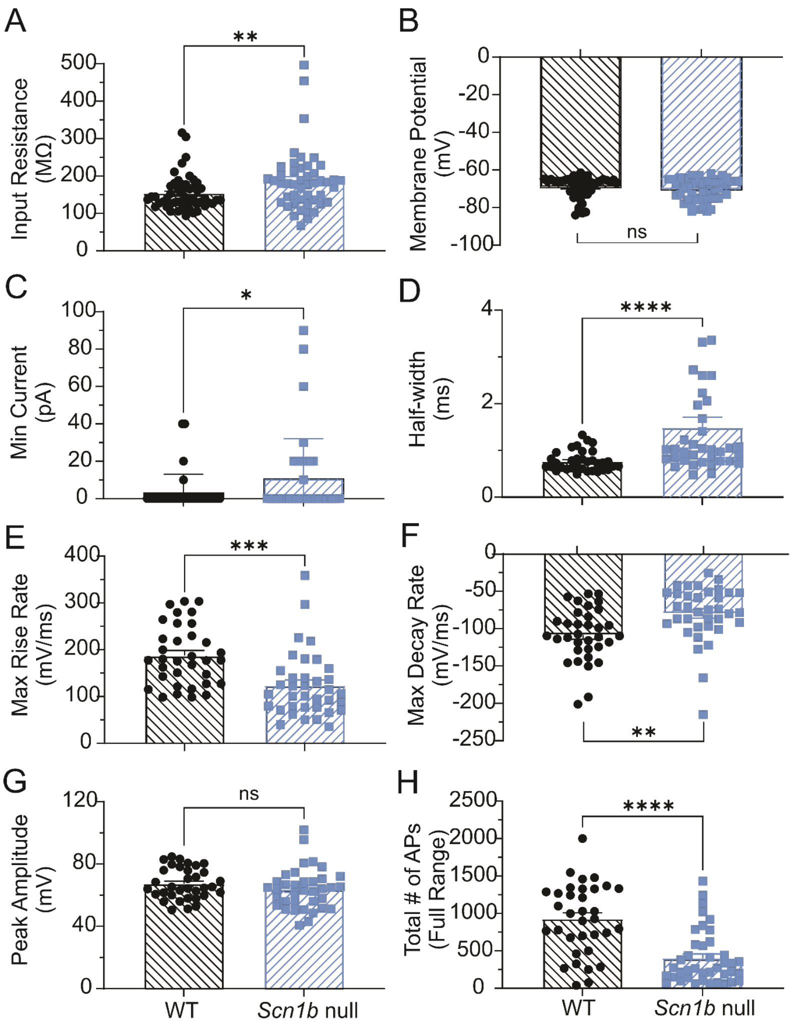

Supplemental Fig. 3. *Scn1b* deletion altered passive and active membrane electrical properties of PCs. A and B. Comparisons of membrane input resistance and resting membrane potentials between WT and *Scn1b* null PCs, respectively. **C and D.** Comparisons of minimum current required to evoke an AP and half amplitude duration of APs in WT or *Scn1b* null PCs, respectively. **E and F.** Comparisons of maximum rise and decay rates of APs of WT or *Scn1b* null PCs, respectively. **G.** Comparisons of the peak amplitude of AP firing of WT or *Scn1b* null PCs. **H.** Comparisons of total # of APs of WT or *Scn1b* null PCs, respectively. The AP kinetics (Half-width, Maximum rise and decay rate and peak amplitude were calculated by averaging the first 5 APs evoked by 80 pA current injection. The total # of APs is sum of all APs evoked by current injections from -60 pA to 180 pA. Values are mean ± SEM of 34-72 cells from 20-30 WT mice or *Scn1b* null mice, respectively. Asterisks indicate significant differences between WT and *Scn1b* null PCs (* *p<0.05,* ** *p<0.01,* *** *p<0.005,* **** *p<0.0001*).

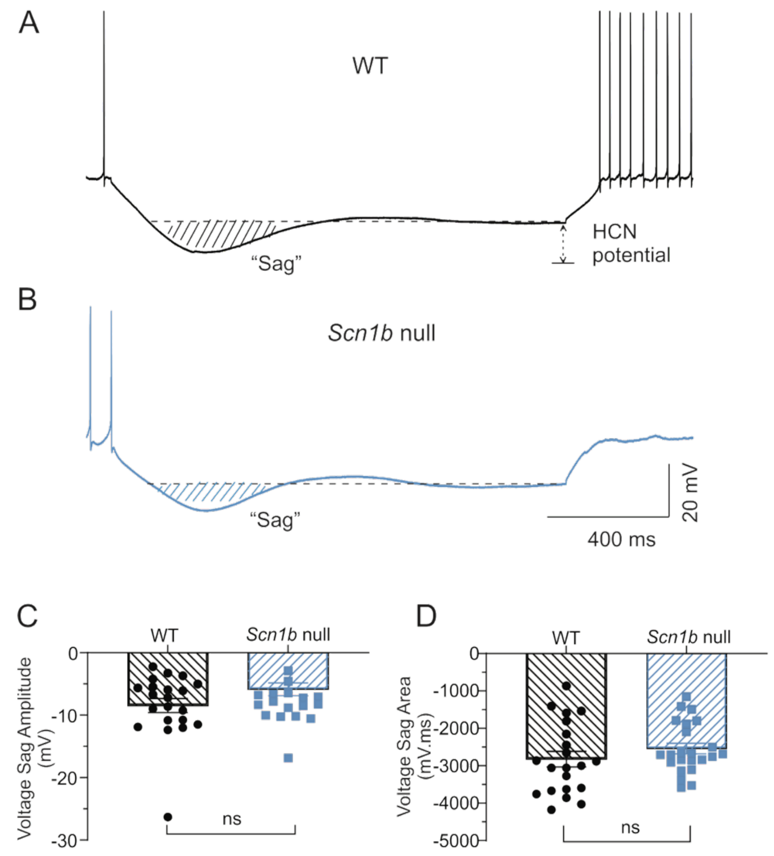

Supplemental Fig. 4. *Scn1b* deletion does not affect hyperpolarization-activated cyclic nucleotide-gated (HCN) channel activity. A and B. HCN channel-mediated potential changes are expressed as voltage Sag (below the dotted lines). Traces show injection of -60 pA current-evoked responses recorded from a WT (black, A) or *Scn1b* null (blue, B) PC. HCN potentials or voltage Sag amplitudes were measured as the maximum membrane hyperpolarization minus the steady state potential baseline just before offset of the hyperpolarizing current injection pulse as indicated by the arrow in A, whereas the area was measured accordingly as indicated by the shaded areas in A and B. **C and D.** HCN potentials (C) or voltage sag areas (D) compared between WT and null PCs. Values are Mean ± SEM of 21 PCs from 16 WT mice or 23 PCs from 17 *Scn1b* null mice, respectively.

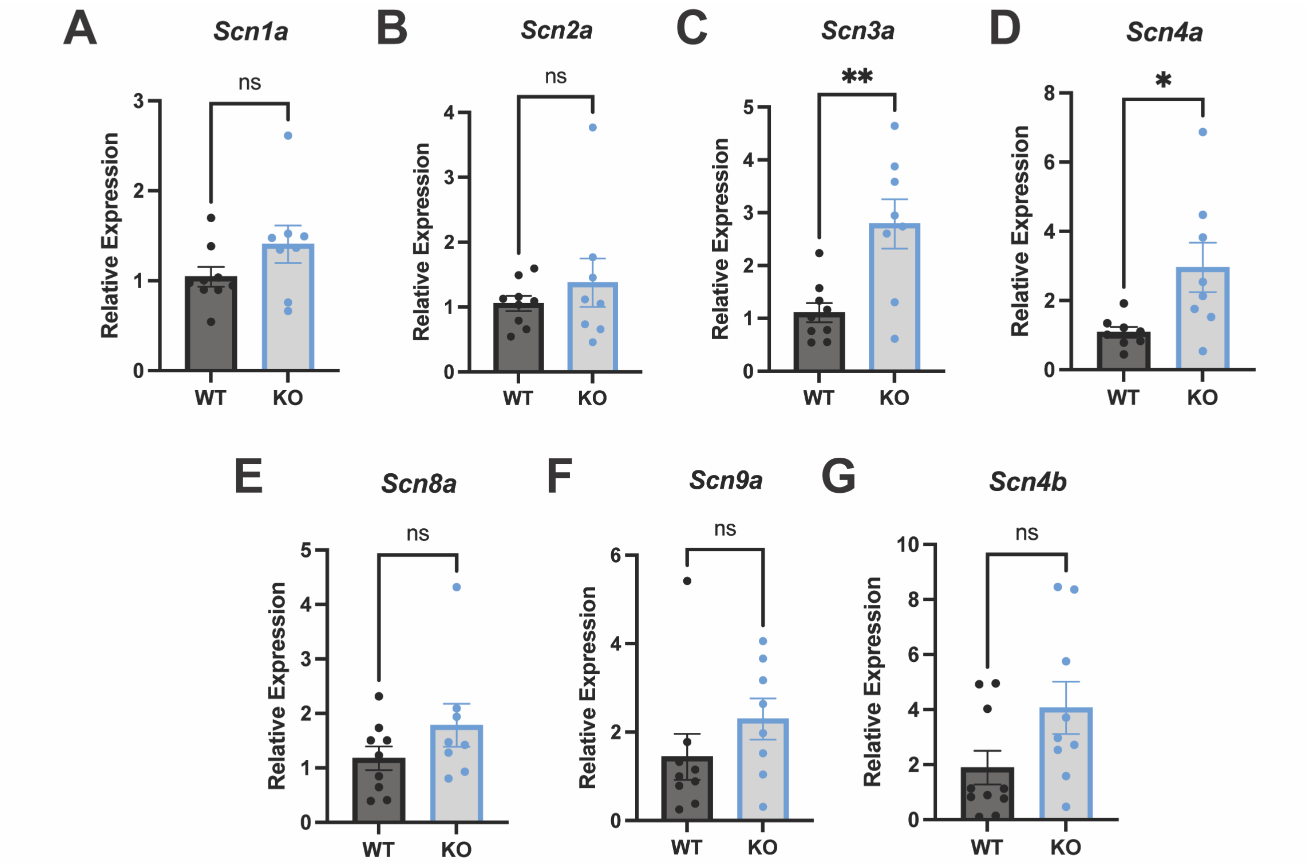

Supplemental Fig. 5. mRNA abundance in P15-17 *Scn1b* null vs WT cerebellum. RT-qPCR of *Scn1a* (A), *Scn2a* (B), *Scn3a* (C), *Scn4a* (D), *Scn8a* (E), *Scn9a* (F), and *Scn4b* (G) between WT (black) and null (blue) cerebellum. *Scn3a* (C) and *Scn4a* (D) were significantly increased in *Scn1b* null (blue) compared to WT (black) cerebellum. Statistical significance was determined using unpaired Student's t-test (* : P < 0.05; **: P < 0.01). Data are represented as the mean ± SEM. WT: n = 8-9, null: n = 8. Male and female pups were used in all experiments.

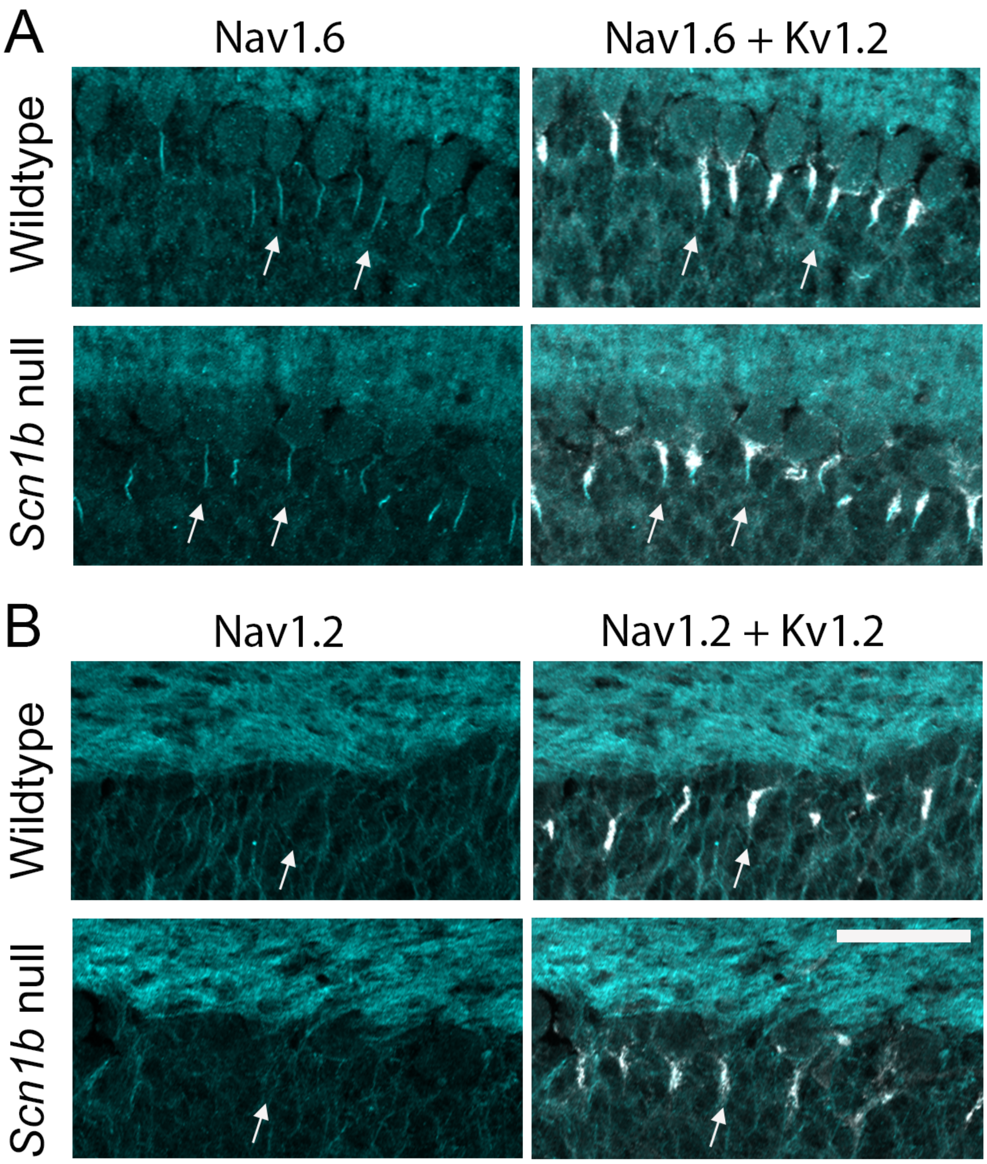

Supplemental Fig. 6. VGSC expression in *Scn1b* null Purkinje neuron AISs and in the molecular layer is comparable to WT. A. Representative images of Nav1.6 expression in P17 WT and *Scn1b* null cerebellum, showing dense labeling in the molecular layer and at PC AISs. Cyan: Nav1.6, grey: Kv1.2. **B.** Representative images of Nav1.2 expression in WT and *Scn1b* null cerebellum, showing dense labeling in the molecular layer but not at AISs. Cyan: Nav1.2, grey: Kv1.2. Scale bar: 25 μm.

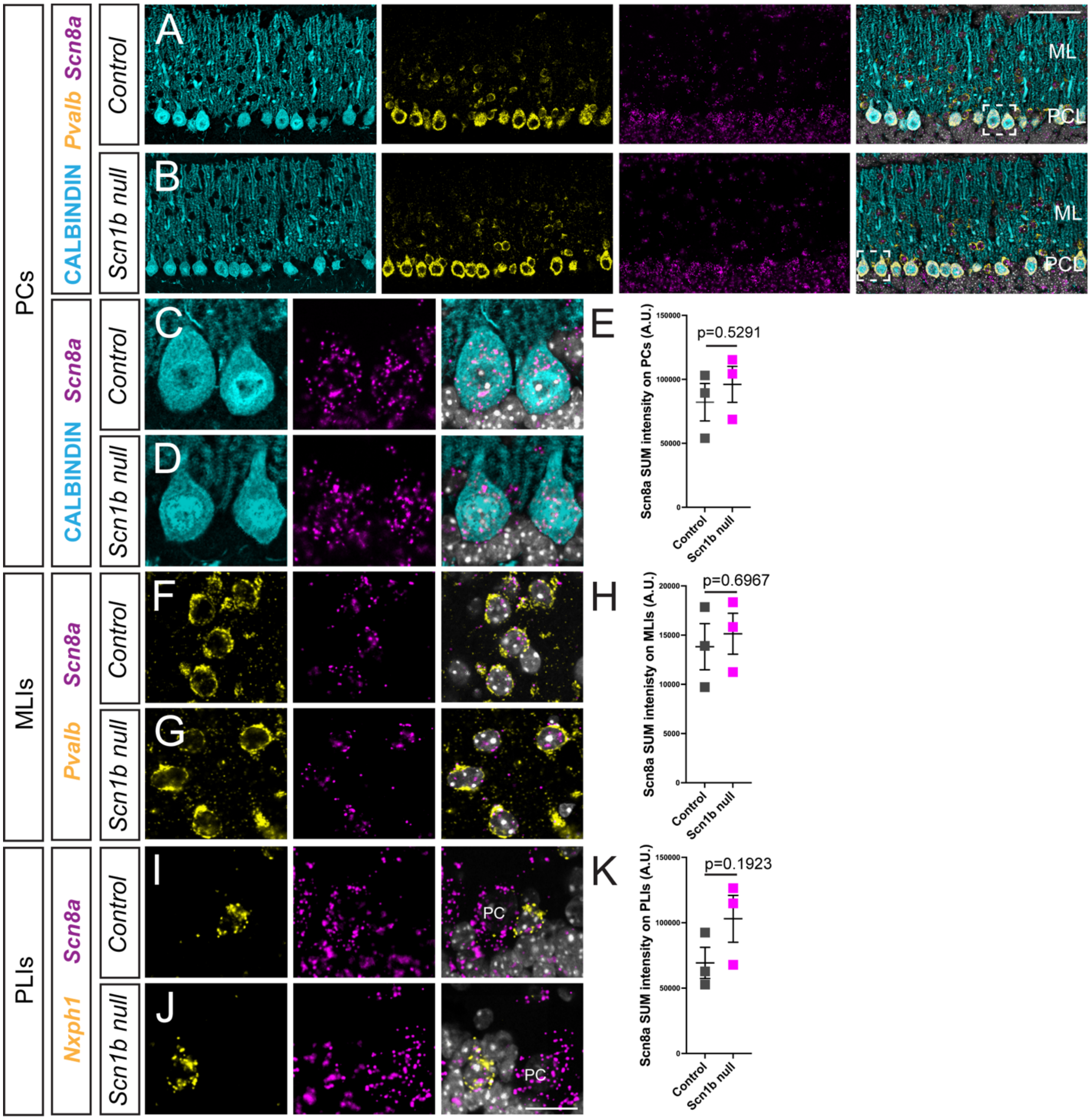

Supplemental Fig. 7. ***Scn8a*** **RNAscope signal levels are comparable in PCs, MLIs, and PLIs of WT and** ***Scn1b*** **null cerebellum. A-D**. IHC-RNAscope stain for anti-calbindin (CALBINDIN)+*Pvalb*+*Scn8a* shows that *Scn8a* signal levels are comparable in PCs of control (WT) and *Scn1b* null cerebellum (**E**). **F, G.** RNAscope stain for *Pvalb*+*Scn8a* shows that *Scn8a* signal levels are comparable in MLIs of control (WT) and *Scn1b* null cerebellum (**H**). **I, J**. RNAscope stain for *Nxph1*+*Scn8a* shows that *Scn8a* signal levels are comparable in PLIs of Control (WT) and *Scn1b* null cerebellum (**K**). ML: Molecular layer. PCL: Purkinje Cell Layer. Dotted boxes in A, B show the location of C, D. Data were analyzed using unpaired t-test, two-tailed.

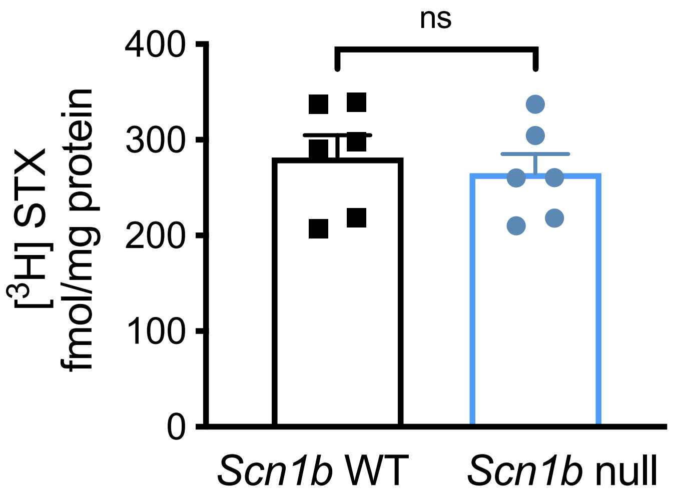

Supplemental Fig. 8. ^{3}H-STX binding analysis of *Scn1b* WT and null cerebellar brain membrane preparations. Cerebellar membranes were prepared from P16-18 WT and *Scn1b* null mice as described in Methods. No difference in total levels of TTX-S VGSC protein was measured between genotypes. Black: WT, Blue: null. ns: not significant, unpaired t-test.

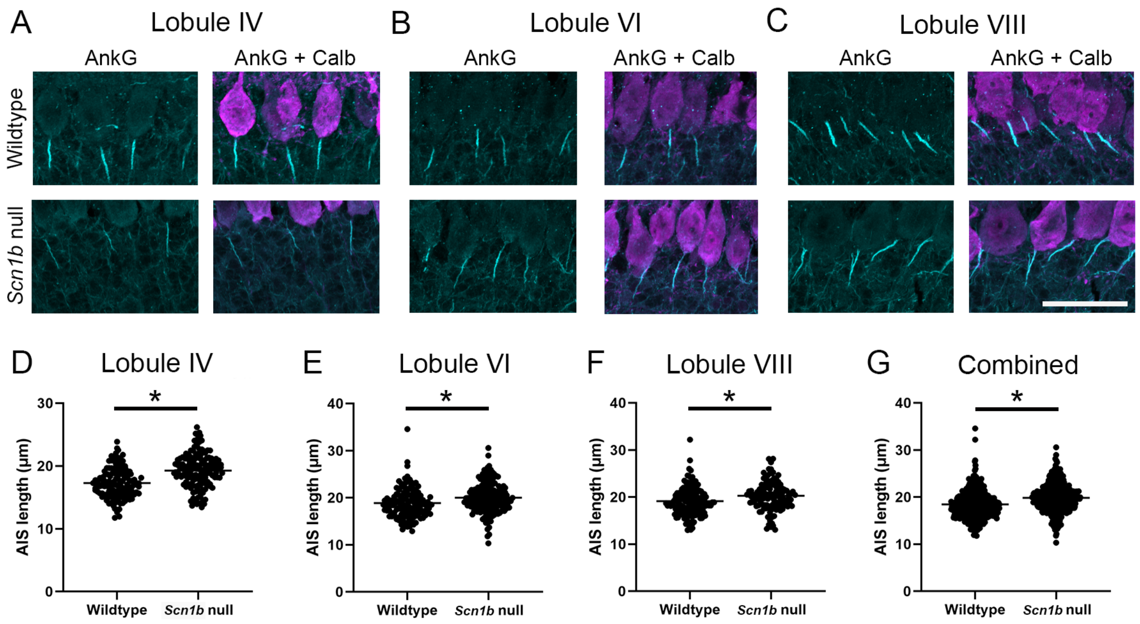

Supplemental Fig. 9. PC AISs in *Scn1b* null mice are longer than in WT. A-C. Representative images of PC AISs in cerebellar lobule IV (A), lobule VI (B), and lobule VIII (C), with AnkG in cyan to mark AISs and calbindin in magenta to mark PCs in P17 mice. Top: WT; bottom: *Scn1b* null. **D-G**. Scatter plot graphs displaying quantification of AIS length in WT and *Scn1b* null in lobule IV (D), lobule VI (E), lobule VIII (F), and in all three lobules combined (G). * : $P < 0.01$, unpaired t-test, two-tailed. Scalebar: 50 µm.

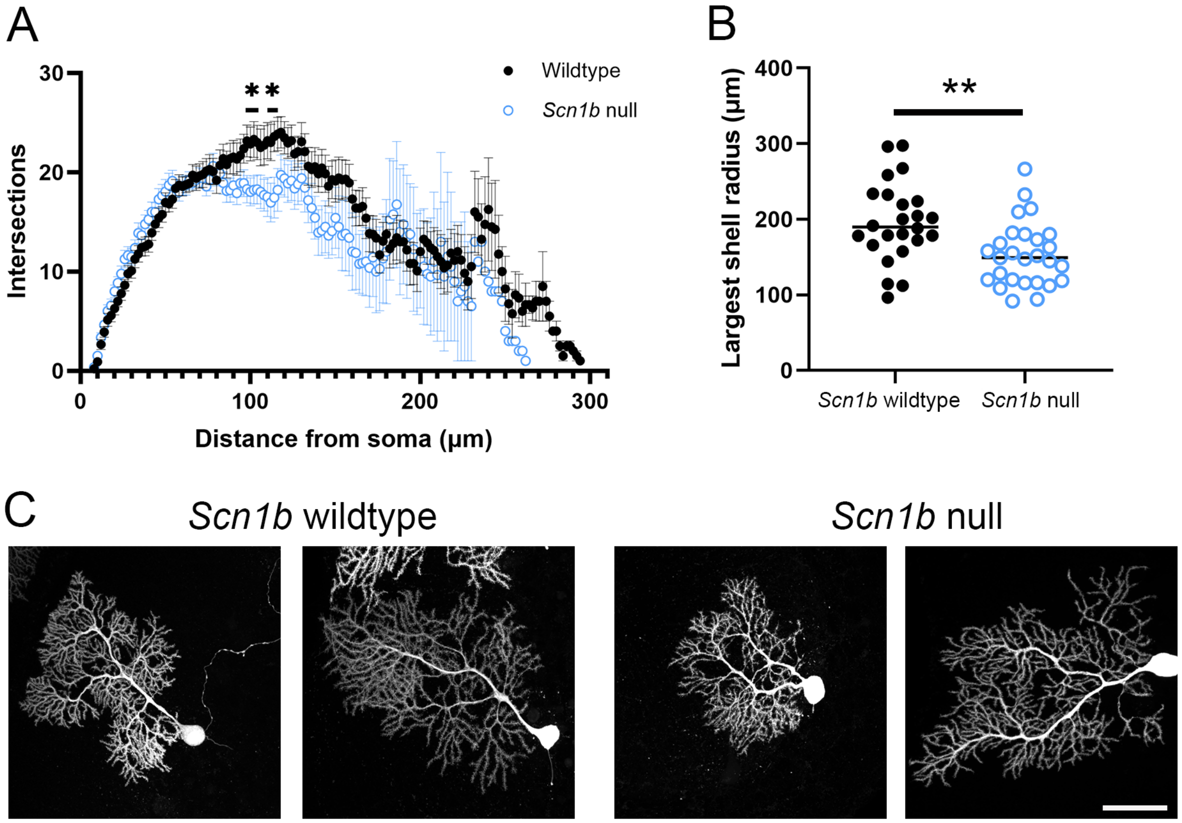

Supplemental Fig. 10. Sholl analysis of *Scn1b* null and WT PCs. A. Sholl analysis of PC dendritic arborization in *Scn1b* WT and null mice showing number of intersections vs distance from soma. Statistical differences between genotypes are indicated. **B.** PC radii are larger in WT cerebellum than in *Scn1b* null. **C.** Representative images of *Scn1b* WT and null Purkinje neurons. **: P < 0.005, unpaired t-test. Scalebar: 50 µm.

Supplemental Tables

	**WT**	***Scn1b* null**
Stride Length (mm):		
Forepaw	38.25 ± 1.51	35.03 ± 1.00
Hindpaw	38.36 ± 1.36	34.31 ± 1.32
Stride Width (mm):		
Forepaw	11.25 ± 0.38	17.50 ± 1.00 **
Hindpaw	18.94 ± 0.86	20.59 ± 0.85
Step Angle (°):		
Forepaw	116.75 ± 3.08	87.75 ± 3.39 **
Hindpaw	89.61 ± 3.72	79.25 ± 2.93 *
Ratio of Stride Width	0.60 ± 0.02	0.85 ± 0.03***
Ratio of Stride Angle	1.31 ± 0.028	1.11 ± 0.23***
Femur Length (cm)	9.79 ± 0.14	9.04 ± 0.23 *
Humerus Length (cm)	8.67 ± 0.06	8.20 ± 0.14 *
Relative Forepaw Stride Width	1.00 ± 0.03	1.64 ± 0.09
Relative Hindpaw Stride Width	1.00 ± 0.05	1.18 ± 0.05

Supplemental Table 1. Gait analysis parameters. *: $P < 0.05$, **: $P < 0.005$, ***: $P < 0.001$

Parameter	WT	*Scn1b* Null
Resting Membrane Potential (mV)	-68.94 ± 0.88 (39)	-70.95 ± 0.93 (41)
Capacitance (pF)	27.70 ± 2.89 (69)	28.73 ± 3.75 (64)
Membrane Input Resistance (mΩ)	145.12 ± 6.35 (48)	201.75 ± 24.78* (43)
Minimum Current Required (pA)	3.00 ± 1.81 (31)	8.00 ± 3.00* (26)
Maximum Evoked AP Firing Frequency (Hz)	48.84 ± 3.68 (30)	29.23 ± 3.39**** (27)
Peak AP Amplitude (mV)	68.39 ± 2.54 (19)	61.52 ± 2.56* (19)
AP Half-Width (ms)	0.73 ± 0.04 (19)	1.34 ± 0.17** (19)
Maximum AP Rise Rate (mV/ms)	202.09 ± 16.16 (19)	113.25 ± 16.63*** (19)
Maximum AP Decay Rate (mV/ms)	-118.44 ± 9.62 (19)	-77.79 ± 9.58** (19)
% of cells firing spontaneously	87% (33/38)	50% (25/50)
% of cells with bursting	5.00 (2/38)	30.00 (15/50)
% of cells firing < 3 APs	19.51 (8/41)	44.00 (22/50)

Supplemental Table 2. Passive and active membrane electric properties of cerebellar PCs. *, P<0.05; **, P<0.01; ***, P< 0.001; ****, P< 0.0001

	$V_{1/2}$ Activation (mV)	$V_{1/2}$ Inactivation (mV)	Activation Slope factor (mV^{-1})	Inactivation Slope factor (mV^{-1})
WT (n=12)	-40.0 ± 1.3	-64.1 ± 1.0	6.36 ± 0.4	-5.25 ± 0.1
Scn1b null (n=13)	-40.7 ± 1.0	-67.7 ± 1.3*	6.78 ± 0.3	-5.74 ± 0.1

Supplemental Table 3. Voltage dependent properties of acutely isolated WT and null PCs.

No differences between genotypes, Unpaired t-test with $P < 0.05$.

Antigen	Source	Host Species	Dilution
Nav1.6	Alomone, cat.# ASC-009	Rabbit	1:250
Nav1.2	Alomone, cat.# ASC-002	Mouse	1:250
Kv1.2	Antibodies Inc, cat.# 75-008	Mouse	1:250
V5	Cell Signaling Technology, cat.# 13202	Rabbit	1:500
AnkyrinG	Gift, Dr. Paul Jenkins PMC11097922	Goat	1:500
Anti-rabbit IgG (H+L), AlexaFluor 488 or 568	Invitrogen, 488 = A-11008, 568 = A-11036	Goat	1:500
Anti-mouse IgG (H+L), AlexaFluor 488 or 568	Invitrogen, 488 = A-11029, 568 = A-11004	Goat	1:500

Supplemental Table 4. Primary antibodies used for immunofluorescence labeling.

Supplemental Videos:

Supplemental Video 1. Compiled video recordings from a top-down view showing three wildtype and three *Scn1b* null mice walking freely. Additional footage of *Scn1b* gait is included at the end of the video.

Supplemental Video 2. Compiled video recordings from a side view showing three wildtype and three *Scn1b* null mice walking freely.
